# Expected Shannon Entropy and Shannon Differentiation between Subpopulations for Neutral Genes under the Finite Island Model

**DOI:** 10.1371/journal.pone.0125471

**Published:** 2015-06-11

**Authors:** Anne Chao, Lou Jost, T. C. Hsieh, K. H. Ma, William B. Sherwin, Lee Ann Rollins

**Affiliations:** 1 Institute of Statistics, National Tsing Hua University, Hsin-Chu, Taiwan; 2 EcoMinga Foundation, Via a Runtun, Baños, Tungurahua, Ecuador; 3 Evolution & Ecology Research Centre, School of Biological Earth and Environmental Science, The University of New South Wales, Sydney, New South Wales, Australia; 4 Cetacean Research Unit, Murdoch University, South Road, Murdoch, Western Australia, Australia; 5 Centre for Integrative Ecology, School of Life and Environmental Sciences, Deakin University, Geelong, Victoria, Australia; University of South Australia, AUSTRALIA

## Abstract

Shannon entropy *H* and related measures are increasingly used in molecular ecology and population genetics because (1) unlike measures based on heterozygosity or allele number, these measures weigh alleles in proportion to their population fraction, thus capturing a previously-ignored aspect of allele frequency distributions that may be important in many applications; (2) these measures connect directly to the rich predictive mathematics of information theory; (3) Shannon entropy is completely additive and has an explicitly hierarchical nature; and (4) Shannon entropy-based differentiation measures obey strong monotonicity properties that heterozygosity-based measures lack. We derive simple new expressions for the expected values of the Shannon entropy of the equilibrium allele distribution at a neutral locus in a single isolated population under two models of mutation: the infinite allele model and the stepwise mutation model. Surprisingly, this complex stochastic system for each model has an entropy expressable as a simple combination of well-known mathematical functions. Moreover, entropy- and heterozygosity-based measures for each model are linked by simple relationships that are shown by simulations to be approximately valid even far from equilibrium. We also identify a bridge between the two models of mutation. We apply our approach to subdivided populations which follow the finite island model, obtaining the Shannon entropy of the equilibrium allele distributions of the subpopulations and of the total population. We also derive the expected mutual information and normalized mutual information (“Shannon differentiation”) between subpopulations at equilibrium, and identify the model parameters that determine them. We apply our measures to data from the common starling (*Sturnus vulgaris*) in Australia. Our measures provide a test for neutrality that is robust to violations of equilibrium assumptions, as verified on real world data from starlings.

## Introduction

Genetic analysis of populations has nearly always relied on measures based on expected heterozygosities or gene identities [1], because these link to variance and the binary nature of sexual reproduction and diploid inheritance. The corresponding *F*_*ST*_ measures and their various generalizations for subdivided populations have also played a central role in population genetics and evolutionary biology [2,3,4]. This approach emphasizes the frequent alleles by giving them much more weight than their population fraction, and multi-level hierarchical additive partitioning is not usually possible with heterozygosity-based measures [5–8].

Researchers in various disciplines have increasingly recognized that diversity within populations and compositional differentiation between populations cannot be completely characterized by a single measure. For example, ecologists have reached a consensus [9,10] that instead of one or a few diversity measures, it is best to use a multifaceted diversity measure parameterized by order *q* (which determines the measures’ emphasis on rare or common species), to completely characterize the species abundance distributions in ecological assemblages. By analogy, in addition to measures based on heterozygosity, complementary abundance-sensitive measures that are sensitive to less frequent alleles are needed to portray a more complete picture of allele frequency distribution or differentiation among populations.

This paper mainly focuses on Shannon entropy *H* and its differentiation measures. Shannon entropy *H* and its monotonic transformations, such as exp(*H*), connect directly to the rich mathematics of information theory initiated by Shannon [11], singularly appropriate for DNA information [12,13,14]. Unlike heterozygosity, information measures weigh alleles in proportion to their population fraction. Shannon entropy and its exponential are also the most popular summary statistics for ecological biodiversity [15], so their use in genetics would allow integrated ecological and genetic modeling.

Shannon entropy and its monotonic transformations can be partitioned into independent within- and between-subpopulation components. The between-group component, called mutual information, measures the differentiation of allele proportions between subpopulations as the mean reduction in uncertainty about allele identity when we learn the subpopulation from which the allele was drawn. In measuring compositional differentiation among subpopulations, the between-group component of Shannon entropy obeys stronger monotonicity properties than the between-group component of heterozygosity [8,16] (see Discussion). Mutual information is closely related to entropy-based measures of compositional differentiation among ecological communities [17,18].

Although entropy and mutual information have been widely used in information science and ecology after Shannon [11] and MacArthur [19], they were rarely applied to genetics until recently. Lewontin [20] pioneered the use of entropy and its decomposition in population genetics. Shannon entropy and mutual information have more recently been used to analyze a wide variety of genetic processes and patterns [12,13]. Examples cover a range of taxa, including viruses [21], bacteria [22], protist parasites [23], mosses [24], higher plants [25–31], invertebrates [14,32] and vertebrates including humans [33,34,35]. Many concentrate on microsatellites [12], but they have also assessed AFLPs [29], and single-nucleotide polymorphisms [14]. Recent theoretical uses of Shannon entropy and mutual information in genetics also include: dynamics of populations of genetically variable individuals in landscapes [36]; dynamics of molecules in gene expression networks [37,38,39]; analysis of gene-environment interactions, including genome wide association studies [40–44]; phylogenetic reconstruction [45,46,47]; mapping genes [48,49]; and derivations of classical population genetic results regarding drift and selection [50]. Outside genetics, there is much parallel work in species, phylogenetic and functional diversity involving entropy [51–54], so there may be further opportunities for expansion.

Given all these applications, it is vital to link Shannon entropy and mutual information to neutral genetic models. Previous attempts [12,14,55] fell short of general analytic expressions. For a single isolated population, Sherwin et al. [12] used the diffusion approximation to predict equilibrium Shannon entropy under the infinite allele model (IAM) or stepwise mutation model (SMM). However, these led to slowly-converging infinite series. For two populations connected by dispersal, with SMM, simulation results provided an empirical equation for mutual information at equilibrium, but no analytical equation was obtained [12]. Dewar et al. [14] derived a Taylor approximation to mutual information for bi-allelic genes only. Even with this incomplete armory of methods, Sherwin et al. [12] and Sherwin [13] showed firstly that for analysis of geographic subdivision and genetic exchange between sub-populations, mutual information readily yields an estimate of the dispersal rate per generation, and secondly that compared to all other approaches for analyzing such data, this method is robust to an extraordinarily wide range of dispersal rates and population sizes. The method has been used to assess current and historical subdivision in rainforest trees [25]. Thus mutual information might be more useful than heterozygosity-based measures for genetic estimation of dispersal, as noted by [12,13]. These considerations motivated us to derive analytic formulas for the general case of Shannon entropy and mutual information for genetic data.

Here we report remarkably simple expressions for expected Shannon entropy (and its exponential, “Shannon diversity” or the “effective number of alleles”) of the equilibrium allele distribution at a neutral locus in an isolated population under IAM or SMM. A bridge that connects the two models of mutation is identified. Our formulas and simulations also show for each model a robust relationship between entropy and heterozygosity under neutral models in equilibrium. Simulations show this relationship is often approximately valid even under some non-equilibrium conditions. Thus, the relationship between these two classes of measures may provide a test for neutrality that is relatively robust to violations of equilibrium assumptions.

We generalize this result to find the entropy of subdivided populations that follow the finite island model (FIM), and use the results to predict the mutual information between subpopulations at equilibrium under two models: IAM-FIM (FIM with mutation following IAM) and SMM-FIM (FIM with mutation following SMM). We can thus identify the model parameters that determine mutual information. We apply our measures to common starling (*Sturnus vulgaris*) data collected from their introduced range in Australia, to assess the robustness of the theoretical relationship we have found between entropy and heterozygosity.

## Methods

### Single isolated population under IAM

Assume *N* is the number of diploid individuals in an idealized population, *μ* is the mutation rate per generation, and there are *A* alleles at the target locus, with allele proportions (or fractions) *p*_1_, *p*_2_,…, *p*_*A*_. Throughout the paper, we assume that the population size is sufficiently large so that the distribution of allele proportions is essentially continuous. For non-ideal populations, *N* is replaced by effective population size. Shannon entropy is defined as H1=−∑i=1Apilogpi and heterozygosity is H2=1−∑i=1Api2. Here we use the notation ^1^ *H* for Shannon entropy and ^2^ *H* for heterozygosity because these two measures are special case, of order *q* = 1 and *q* = 2 respectively, of the generalized Tsallis or HCDT entropies ^*q*^ *H* [5,6,7] (see Discussion).

We first seek the expected value of Shannon entropy for neutral alleles under IAM in a single completely isolated population. Using the diffusion approximation, the allele proportion distribution under IAM is approximately Φ(*p*) = *θp* ^−1^(1−*p*)^*θ*−1^, thus the equilibrium expectation value of any function ∑_*i*_ *h*(*p*_*i*_), where *h*(*p*_*i*_) tends to zero when *p*_*i*_ approaches zero, is given by the Ewens’ sampling formula [55]

$$\sum_{i}h(p_{i})\approx \int_{0}^{1}h(p)\Phi (p)dp=\theta \int_{0}^{1}\;h(p)p^{-1}{\left(1-p\right)}^{\theta -1}dp,$$

where *θ* = 4*Nμ*. Setting *h*(*p*) = *p* ^2^ in the above integral, we obtain the well-known formula for the expected heterozygosity [56]:

H2=θ/(θ+1)orθ=[1/(1−H2)]−1. (1)

Setting *h*(*p*) = –*p* log *p*, we obtain the equilibrium expectation of Shannon entropy [12,55]:

H1=−θ∫01(1−p)θ−1logpdp.

The above can be expressed as an integral of the logarithm function with respect to a beta distribution, so we obtain a simple formula for the expected Shannon entropy as a function of *θ* (see S1 Appendix for details)

H1=ψ(θ+1)−ψ(1)=ψ(θ+1)+γ, (2A)

where *ψ*(*z*) is the digamma function, and $\gamma =-\psi (1)=\underset{k\to \infty }{\lim}(\sum_{j=1}^{k}\frac{1}{j}-\log k)$ ≈ 0.5772 is the famous Euler’s constant. It is remarkable that this complex stochastic system has an entropy expressable as a simple combination of well-known mathematical functions. If *θ* is greater than 2, then *ψ*(*θ*+1) can be accurately approximated by log(*θ*+0.5), so for many practical cases the expected Shannon entropy is approximately a linear function of the logarithm of *θ*:

H1≈log(θ+0.5)+0.5772. (2B)

Substituting Eq 1 into Eq 2A or 2B leads to a direct relationship (or link) between expected Shannon entropy and heterozygosity at equilibrium:

H1=ψ[1/(1−H2)]+0.5772≈log[1/(1−H2)−0.5]+0.5772. (3A)

Shannon entropy (^1^ *H*) and heterozygosity (^2^ *H*), can be transformed into an effective number of alleles (or diversity), ^1^ *D* and ^2^ *D*, which possess useful mathematical properties [8,57,58]. The transformation for heterozygosity is ^2^ *D* = 1/(1−^2^ *H*) = *θ* + 1, which is interpreted as the number of equi-frequent alleles that would give the same heterozygosity as that of the actual population. The transformation for Shannon entropy is ^1^ *D* = exp(^1^ *H*), which is interpreted as the number of equi-frequent alleles that would give the same Shannon entropy as that of the actual population [19,56].

We summarize all results for Shannon entropy (*q* = 1, Eq 2A) and heterozygosity (*q* = 2) in the second column of Table 1. When *θ* is greater than 2, the approximation (Eq 2B) leads to the following linear relationship between the Shannon-entropy-based and heterozygosity-based diversities:

D1≈e0.5772(θ+0.5)=1.781(D2−0.5). (3B)

**Table 1:** The expected Shannon entropy ^1^ *H*, heterozygosity ^2^ *H*, for the equilibrium allele distribution at a neutral locus under IAM and SMM for an isolated population, and for a total population (subscript *T*) composed of *n* subpopulations (subscript *S*).

Model/measure	Isolated population	Total population	Subpopulation
IAM:			
Shannon entropy	^1^ *H* = *ψ*(*θ*+1)−*ψ*(1)	^1^ *H*_*T*_ = *ψ*(*θ*_*T*_+1)−*ψ*(1)	H1S=ψ[4N(m*+μ)+1]−∫01ψ(4Nm*y+1)θT(1−y)θT−1dy
			(See S2 Appendix for approximation)
Heterozygosity	^2^ *H* = *θ*/(1+*θ*)	H2T=θT/(1+θT)=1−(4Nnμ+m*+nμm*+μ)−1	H2S=1−4Nm*(1−H2T)+14N(m*+μ)+1 $=1-{\left(4Nn\mu \frac{m^{*}+\mu }{m^{*}+n\mu }+1\right)}^{-1}$
SMM:			
Shannon entropy	^1^ *H* = *ψ*(*θ*+*α*+1)−*ψ*(*α*+1)	^1^ *H*_*T*_ = *ψ*(*θ*_*T*_+*α*_*T*_+1)−*ψ*(*α*_*T*_+1)	H1S=ψ[4N(m*+μ)+αS+1]−∫01ψ(4Nm*y+αS+1)B(αT+1,θT)yαT(1−y)θT−1dy
			(See S3 Appendix for approximation)
Heterozygosity	H2=θα+θ+1=1−1(1+2θ)1/2	H2T=θTαT+θT+1=1−1(1+2θT)1/2	H2S=1−4Nm*(1−H2T)+αS+14Nm*+4Nμ+αS+1

*N* = population size, *m* = dispersal rate, *μ* = mutation rate, *m* ^*^ = *nm*/(*n*–1), *N*_*T*_ = effective population size in the total population, and *ψ*(*x*) = digamma function. See S1 and S2 Appendices for all derivations. For an isolated population, when *α* tends to 0, all formulas for SMM reduce to those for IAM. For the total population, when *α*_*T*_ tend to 0, all formulas for SMM reduce to those for IAM. For subpopulation, when both *α*_*T*_ and *α*_*S*_ tend to 0, all formulas for SMM reduce to those for IAM.

(Notation for IAM) *θ* = 4*Nμ*, $\theta_{T}=4N_{T}\mu =4Nn\mu +\frac{(n-1)\mu }{m^{*}+\mu }$.

(Notation for SMM) *θ* = 4*Nμ*, α = [(1 + 2θ)^1/2^−1]/2, *θ*_*T*_ = [1/(1−^2^ *H*_*T*_)^2^−1]/2, *α*_*T*_ = [1/(1−^2^ *H*_*T*_)−1]/2 = [(1+2*θ*_*T*_)^1/2^−1]/2. αS=4(Nm*)(H2T−H2S)H2S+4(Nμ)(1−H2S)H2S−1, where ^2^ *H*_*T*_ and ^2^ *H*_*S*_ are shown in Eqs 8A and 8B. *B*(*x*,*y*) = Γ(*x*)Γ(*y*)/Γ(*x*+*y*): beta function, Γ(*x*): gamma function.

In this regime the Shannon diversity is itself a linear function of *θ*.

### Single isolated population under SMM

Ohta and Kimura developed the framework of SMM, in which each mutation only creates adjacent alleles [59,60,61]. Here we consider the simplest form: the one-phase mutation model in which mutation is always only a single step, e.g. to one more or less repeat in microsatellite DNA. They used a diffusion approximation to obtain the allele proportion distribution:

$$\Phi (p)=\frac{{\left(1-p\right)}^{\theta -1}\;p^{\alpha -1}}{B(\alpha +1,\theta )},$$

where *θ* = 4*Nμ*, *α* = [(1+2*θ*)^1/2^−1]/2, *B*(*x*,*y*) = Γ(*x*)Γ(*y*)/Γ(*x*+*y*) is the beta function, and Γ(*x*) is the gamma function. Their approach in [60] is reviewed in S1 Appendix to provide the necessary background for the generalization to the theory of multiple populations. As implied by their theory and also explained in S1 Appendix, if the parameter *α* tends to 0, then the allele distribution tends to that in IAM. This explicitly bridges between the allele proportion distributions of SMM and IAM, implying all properties derived from allele proportion distributions of the two models can also be connected by this bridge. For example, the expected heterozygosity ^2^ *H* derived by Kimura & Ohta is [60]:

H2=θα+θ+1. (4A)

When *α* is zero, the above reduces to the expected heterozygosity under IAM (in Eq 1). Using the relationship between *α* and *θ* (*α* = [(1+2*θ*)^1/2^−1]/2; details in S1 Appendix), we can also express the expected heterozygosity in terms of a function of only *θ*:

H2=1−1(1+2θ)1/2. (4B)

From the allele proportion distribution, the expected Shannon entropy for a population in mutation-drift equilibrium under SMM is approximately equal to

H1≈∫01(−plogp)Φ(p)dp=∫01(−logp)pα(1−p)θ−1B(α+1,θ)dp.

Again, this is the negative of an integral of the logarithm function with respect to a beta distribution. We thus have a simple analytic formula for expected Shannon entropy under SMM: (see S1 Appendix for derivation of the following three equations):

H1=ψ(α+θ+1)−ψ(α+1). (5A)

When *α* is zero, the above reduces to the expected Shannon entropy under IAM (Eq 2A). From Eqs 4A, 4B and 5A, we obtain a simple relationship (or link) between ^1^ *H* and ^2^ *H* (see S1 Appendix for details):

H1≈log(1+H2−(H2)21−H2), (5B)

and between ^1^ *D* and ^2^ *D*:

D1≈1+D2−1D2. (5C)

We summarize all results for Shannon entropy (*q* = 1, Eq 5A) and heterozygosity (*q* = 2, Eqs 4A or 4B) in the second column of Table 1.

### Multiple populations under IAM-FIM

In Wright’s finite island model (FIM) there are *n* idealized subpopulations each with size *N*, mutation rate *μ* per generation, and dispersal (or migration) rate *m* per generation, so that in each generation the alleles of any subpopulation include a proportion *m/*(*n−*1) randomly chosen from each of the other *n−*1 subpopulations. For notational simplicity, we follow Latter [62] and use *m* ^*^ = *mn*/(*n*_*—*_1) instead of *m*. Note FIM assumes that population size, dispersal rate and mutation rate are all constant across all subpopulations. Spatially homogeneous dispersal is also assumed [63].

As with a single isolated population, the allele proportion *y* for the total population is [64]:

$$\Phi_{T}(y)=\theta_{T}y^{-1}{\left(1-y\right)}^{\theta_{T}-1},0\leq y\leq 1,$$ (6)

where *θ*_*T*_ = 4*N*_*T*_ *μ* $=4Nn\mu +\frac{(n-1)\mu }{m^{*}+\mu }$, and *N*_*T*_ denotes the effective size of the total population *N*_*T*_ = *Nn*+(*n*−1)/[4(m*+*μ*)] under IAM-FIM ([65], p. 431). Therefore, all formulas for a single isolated population can be used for the total population if the parameter *θ* in a single population is replaced by the effective number of mutations per generation in the total population *θ*_*T*_. We summarize the results in the third column of Table 1.

Barton & Slatkin [66] showed that the conditional distribution for allele proportion *x* in a subpopulation, given its proportion in the total population *y*, can be expressed as:

$$\phi (x|y)=K{\left(1-x\right)}^{4Nm^{*}(1-y)+4N\mu -1}x^{4Nm^{*}y-1},$$

where *K* = 1 / *B*(4*Nm* ^*^ *y* +1, 4*Nm* ^*^(1_—_ *y*)+4*Nμ*), a normalizing constant so that ∫xϕ(x|y)dx=1, and *B* is a beta function defined earlier. The unconditional proportion *x* can be obtained by integrating over all possible *y* values in the total population with distribution function given in Eq 6. Then the allele proportional distribution in a subpopulation is

$$\Phi_{S}(x)=\int_{0}^{1}\phi (x|y)y\Phi_{T}(y)dy=\int_{0}^{1}K\;x^{4Nm^{*}y-1}{\left(1-x\right)}^{4Nm^{*}(1-y)+4N\mu -1}\theta_{T}{\left(1-y\right)}^{\theta_{T}-1}dy.$$ (7A)

Based on the above distribution, we can directly obtain the heterozygosity for a subpopulation as

H2S=1−∫01x2ΦS(x)dx=1−∫01(4Nm*y+14N(m*+μ)+1)yΦT(y)dy=1−4Nm*(1−H2T)+14N(m*+μ)+1=1−4Nm*/(θT+1)+14N(m*+μ)+1. (7B)

This formula (Eq 7B) was derived in Maruyama [67], using a recurrence formula for heterozygosity in the total and in a subpopulation; see Rousset [68] for a review. Our approach here is a direct method based on the allele proportion distribution.

Based on the distribution in Eq 7A, the exact formula for Shannon entropy for a subpopulation can be expressed as: (see S2 Appendix).

H1S=−∫01(xlogx)ΦS(x)dx=ψ[4N(m*+μ)+1]−∫01ψ(4Nm*y+1)θT(1−y)θT−1dy. (7C)

The above formula can be numerically evaluated using standard numerical integration software. Table 1 (last column) summarizes the exact formulas for expected subpopulation entropy and heterozygosity. A general approximation in terms of the digamma function is

H1S≈(ψ[4N(m*+μ)+1]−ψ(4Nm*θT+1+1))+12(4Nm*4Nm*+θT+1)2θT(θT+2). (7D)

See S2 Appendix for derivation and for more approximation formulas under various conditions to examine some analytic properties; see Discussion for some special cases.

### Multiple populations under SMM-FIM

Based on the theory of Rousset [68] under SMM-FIM, we can express the expected heterozygosities of the total population and in a subpopulation as follows:

H2T=1−1π∫0π(m*/nμ(1−cost)+1n)(4N(1−cost)μ+m*/nμ(1−cost)+1)−1dt; (8A)

H2S=1−1π∫0π(m*/nμ(1−cost)+1)(4N(1−cost)μ+m*/nμ(1−cost)+1)−1dt. (8B)

Note that if *m* = 0 and *n* = 1, then both heterozygosities in SMM-FIM reduce to that in a single population under the model SMM. That is, in the case *m* = 0, *n* = 1, we have ^2^ *H*_*S*_ = ^2^ *H*_*T*_ = 1−1/(1+8*Nμ*)^1/2^; see Eq 4B.

As derived in S3 Appendix, the allele proportion distribution in the total population can be written as

$$\Phi_{T}^{}(y)=\frac{{\left(1-y\right)}^{\theta_{T}-1}y^{\alpha_{T}-1}}{B(\alpha_{T}+1,\theta_{T})},$$

where *θ*_*T*_ = 4*N*_*T*_ *μ*, *α*_*T*_ = [(1+2*θ*_*T*_)^1/2^−1]/2 and *N*_*T*_ is the effective total population size under SMM-FIM. We can express *N*_*T*_ as a formula in terms of *m* and *μ*; see below for description. Comparing the allele proportion distributions of a single isolated population and of the total population of a subdivided population, we see that both have exactly the same form, but the parameters (*α*, *θ*) in an isolated population should be replaced by (*α*_*T*_, *θ*_*T*_) in the subdivided population. Thus, all results in an isolated SMM are also valid for the total population with population parameters (*α*_*T*_, *θ*_*T*_). For example, the expected heterozygosity in the total population can be expressed as ^2^ *H*_*T*_ = 1−1/(1+8*N*_*T*_ *μ*)^1/2^, and *θ*_*T*_ and *α*_*T*_ can be expressed as functions of heterozygosities (see S3 Appendix):

θT=[1/(1−H2T)2−1]/2,αT=[1/(1−H2T)−1]/2. (8C)

Substituting Eq 8A into Eq 8C, we can express *θ*_*T*_ (and thus *N*_*T*_) as well as *α*_*T*_ in terms of *m* and *μ*. Shannon entropy has the same formula as that given in Eqs 4A and 4B, with (*α*, *θ*) replaced by (*α*_*T*_, *θ*_*T*_). Table 1 (with column label “Total population” for the model SMM) summarizes the formula. Note here if *α*_*T*_ tends to 0, then all results reduce to those under IAM. This shows the fundamental connection between IAM and SMM formulas for the total population.

In S3 Appendix, we also derive the allele proportion distribution in a subpopulation. Consider an allele with allele proportion *x* in the subpopulation given its allele proportion in the total population is *y*, and let *ϕ*(*x*|*y*) be the conditional allele frequency distribution. Applying Wright’s formula [69], we obtain the conditional steady-state allele proportion distribution in a subpopulation:

$$\phi (x|y)=K_{S}x^{4Nm^{*}y+\alpha_{S}-1}{\left(1-x\right)}^{4Nm^{*}(1-y)+4N\mu -1},$$ (9A)

where *K*_*S*_ = 1/*B*(4Nm*y + *α*_*S*_ + 1, 4Nm*(1−*y*) + 4*Nμ*) and *α*_*S*_ can be expressed as a function of heterozygosities:

αS=4Nm*(H2T−H2S)H2S+4Nμ(1−H2S)H2S−1.

(It then follows from Eqs 8A and 8B that *α*_*S*_ can be expressed as a function of *m* and *μ*.) Thus we have the marginal allele proportion distribution in a subpopulation:

$$\Phi_{S}(x)=\int_{0}^{1}\phi (x|y)y\Phi_{T}(y)dy=\frac{1}{B(\alpha_{T}+1,\theta_{T})}\int_{0}^{1}K_{S}\;x^{4Nm^{*}y+\alpha_{S}-1}{\left(1-x\right)}^{4Nm^{*}(1-y)+4N\mu -1}y^{\alpha_{T}}{\left(1-y\right)}^{\theta_{T}-1}dy.$$ (9B)

When both *α*_*T*_ and *α*_*S*_ tend to 0, the allele proportion distribution of SMM given in Eq 9B reduce to that of IAM given in Eq 7A. Based on this distribution, the expected heterozygosity of a subpopulation becomes

H2S=1−∫01x2ΦS(x)dx=1−4Nm*(1−H2T)+αS+14Nm*+4Nμ+αS+1.

Also, we obtain the expected Shannon entropy for a subpopulation:

H1S=ψ(4Nm*+4Nμ+αS+1)−∫01ψ(4Nm*y+αS+1)B(αT+1,θT)yαT(1−y)θT−1dy. (9C)

As shown in S3 Appendix, this Shannon entropy for a typical subpopulation can be approximated by:

H1S≈ψ(4Nm*+4Nμ+αS+1)−ψ(4Nm*(αT+1)αT+θT+1+αS+1)+12(4Nm*4Nm*(αT+1)+(αS+1)(αT+θT+1))2θT(αT+1)αT+θT+2. (9D)

When both *α*_*T*_ and *α*_*S*_ tend to 0, Eqs (9C) and (9D) reduce to (7C) and (7D) respectively.

### Shannon differentiation measure

Based on the heterozygosities, the commonly used measure *G*_*ST*_ is expressed as *G*_*ST*_ = (^2^ *H*_*T*_−^2^ *H*_*S*_)/(^2^ *H*_*T*_). Since the value of *G*_*ST*_ is constrained by ^2^ *H*_*S*_, a class of unconstrained *n*-assemblage differentiation measures called 1− *C*_*qn*_ were derived [18,70,71]. This class of differentiation measures is independent of within-group diversity. When *q* = 2, this measure gives Jost’s genetic differentiation measure *D* [58], which is a function of heterozygosities, i.e., *D* = 1–*C*_2*n*_ = (^2^ *H*_*T*_−^2^ *H*_*S*_)/[(1−1 / *n*)(1−^2^ *H*_*S*_)]. We can substitute the expectations for ^2^ *H*_*T*_ and ^2^ *H*_*S*_ (given in Table 1) into the formulas of *G*_*ST*_ and *D* to obtain the resulting measures in terms of the model parameters under IAM-FIM and SMM-FIM.

In the limit as *q* approaches unity, the differentiation measure 1− *C*_*qn*_ yields a function of Shannon entropies which is referred to as Shannon differentiation measure throughout the paper:

Shannondifferention=1−C1n=H1T−H1Slogn. (10)

The numerator ^1^ *H*_*T*_−^1^ *H*_*S*_ is the mutual information (*MI*). Division by log *n* standardizes *MI* onto the unit interval if the *n* subpopulations are equally weighted. In the special case of two subpopulations, Shannon differentiation reduces to Horn’s [17] heterogeneity measure in ecology. Substituting the formulas ^1^ *H*_*T*_ and ^1^ *H*_*S*_ (given in Table 1) into the formula for *MI*, we obtain the Shannon differentiation formulas for IAM-FIM and SMM-FIM. Although the *MI* formulas in both models look complicated, we have provided some simplified formulas for IAM-FIM under some circumstances as summarized below (see Table B in S2 Appendix):

1. When 4*Nm* ^*^ >> 4*Nnμ>>*0, *MI* is approximated by a simple function of 4*Nnμ*, *G*_*ST*_ and Jost’s *D* (Eq. B5 in S2 Appendix), revealing that both 4*N*(*m* ^*^+*μ*) (the main factor which determines *G*_*ST*_) and *m* ^*^/(*nμ*) (the main factor which determines Jost’s *D*) affect Shannon differentiation. If the number of mutations is large enough, the ratio *m* ^*^/(*nμ*) becomes the dominating factor (see Discussion). Here *m* ^*^/(*nμ*) = *m*/[(*n*–1)*μ*] is the familiar scaled immigration rate [72].
2. In the case in which 4*Nm* ^*^ >> 4*Nnμ* and 4*Nnμ* is small, *MI* is a simple function of 4*Nnμ* and Jost’s *D* (Eq. B6 in S2 Appendix). In the extreme case that 4*Nnμ* tends to 0, *MI* approaches 0 and thus Shannon differentiation in this extreme case approaches 0.
3. In the opposite case in which 4*Nnμ>>*4*Nm* ^*^, *MI* is a simple function of *m* ^*^/(*nμ*) (Eq. B7 in S2 Appendix). When *m* ^*^/(*nμ*) tends to 0, *MI* approaches log(*n*) and Shannon differentiation approaches unity.

**Fig 1:**
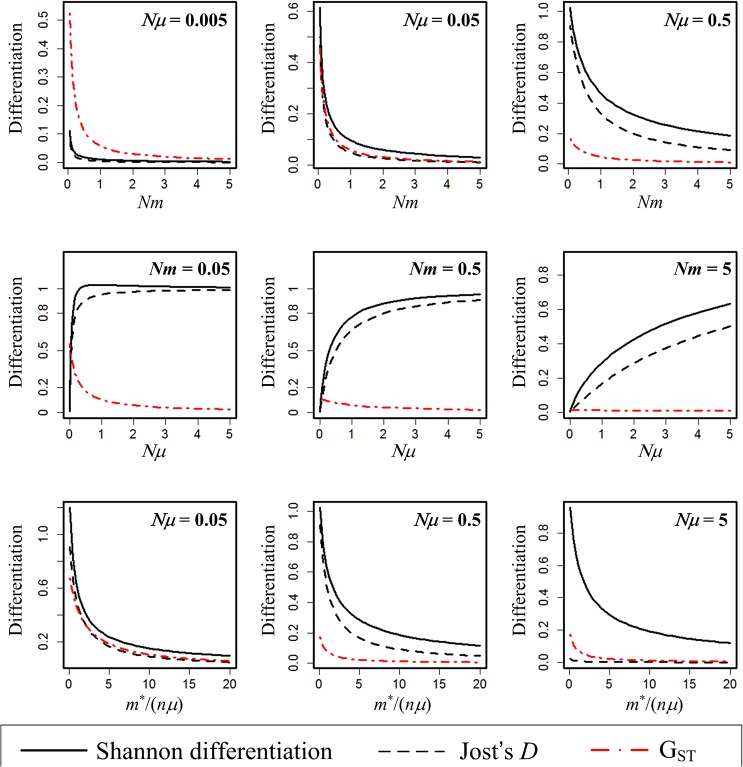
(IAM-FIM *n* = 2, *N* = 5000). Plots of the Shannon differentiation (i.e., normalized mutual information, solid lines), Jost’s differentiation measure *D* (dashed lines), and *G*_*ST*_ (dash-dotted line) as a function of *Nm* (upper panels), *Nμ* (middle panels), and *m* ^*^/(*nμ*) (lower panels).

**Fig 2:**
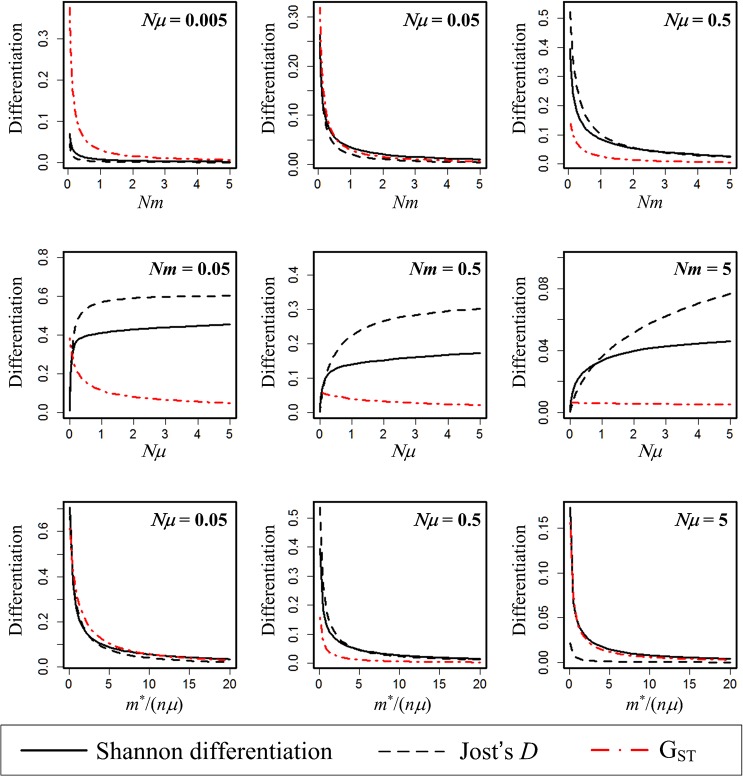
(SMM-FIM *n* = 2, *N* = 5000). Plots of the Shannon differentiation (i.e., normalized mutual information, solid lines), Jost’s differentiation measure *D* (dashed lines), and *G*_*ST*_ (dash-dotted line) as a function of *Nm* (upper panels), *Nμ* (middle panels), and *m* ^*^/(*nμ*) (lower panels).

We plot the performances of *G*_*ST*_, Shannon differentiation, and Jost’s *D* under IAM-FIM (Fig 1) and SMM-FIM (Fig 2) as functions of *Nm* (the average number of dispersals per generation), *Nμ* (the average number of mutations per generation) and *m* ^*^/(*nμ*) (the balance between pairwise dispersal and mutation). The Shannon differentiation measure and Jost’s *D* always exhibit consistent patterns. For both mutation models, the two measures are increasing functions of *Nμ*, and decreasing functions of *Nm* and of *m* ^*^/(*nμ*). Although the classic *G*_*ST*_ measure is also decreasing in *Nm* and in *m* ^*^/(*nμ*), *G*_*ST*_ exhibits a strikingly different pattern being a generally decreasing or stable function of the number of mutations. In the center row of Figs 1 and 2, for Shannon and Jost’s measures: mutation-driven differentiation is more effective when there is low dispersal. In contrast, *G*_*ST*_ is either insensitive to mutation, or at very low mutation rates, the level of differentiation is set by dispersal (compare the three panels of the centre row).

In fact, when *m* > *μ* as in the case of middle right panel in Figs 1 and 2, *G*_*ST*_ becomes nearly independent of *Nμ*, unlike the other two measures. Under IAM-FIM, there is also a dramatic contrast between *G*_*ST*_ and the two measures when 4*Nnμ>>*4*Nm* ^*^ *→* 0 (as the case in the middle left panel of Fig 1). In this case, *MI* approaches log *n*, and thus Shannon differentiation approaches 1, and Jost’s *D* also approaches 1. However, *G*_*ST*_ values are very low and tend to 0 as *Nμ* becomes large. See S2 Appendix for more analytic formulas for mutual information under IAM-FIM.

## Simulation

We did simulations to test the robustness of our predicted relationship between Shannon measures and heterozygosity-based measures. Under IAM, the relationship for an isolated population is given by Eq 3A, ^1^ *H* = ψ[1/(1−^2^ *H*)]+0.5772; this is also valid for the total population under IAM-FIM. Under SMM, Shannon entropy and heterozygosity for an isolated population are linked through the equation ^1^ *H* ≈ log{[1+^2^ *H*−(^2^ *H*)^2^]/(1−^2^ *H*)} (Eq 5B), which holds for the total population under SMM-FIM (Table 1). For a subpopulation, the expected Shannon entropy is a function of not only the expected subpopulation heterozygosity but also the expected total-population heterozygosity. Under IAM-FIM, we propose an explicit link in terms of an integral involving ^2^ *H*_*T*_ and ^2^ *H*_*S*_ (Eq. D7 of S4 Appendix). Under SMM-FIM, the link is not explicit, so numerical procedures are needed; see S4 Appendix for details.

**Fig 3:**
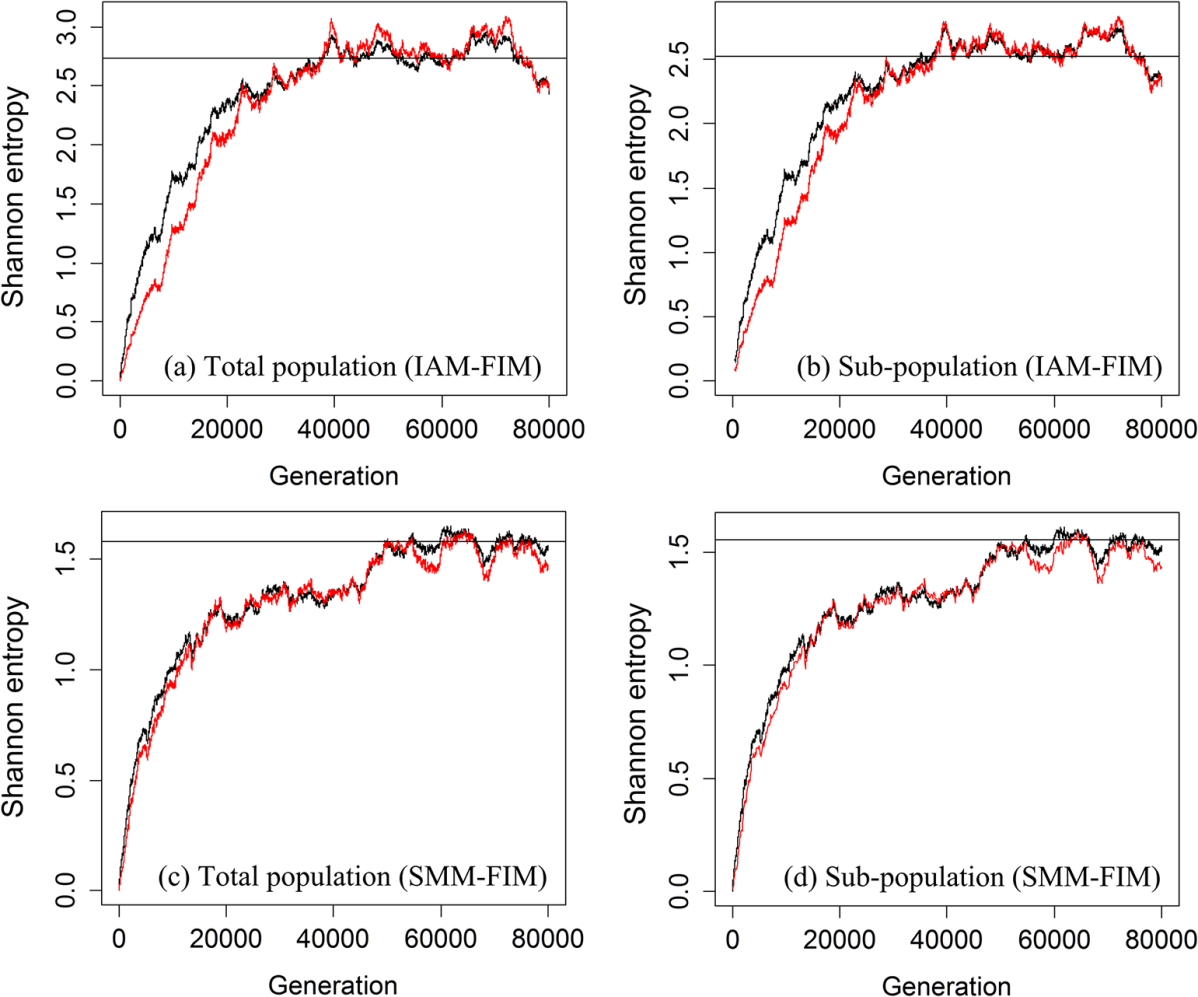
Simulation plots. Simulation results showing stochastic behavior of the average (over 5 loci) of total-population and subpopulation Shannon entropies for *N* = 10000, *n* = 4, *μ* = 0.005%, *m* = 0.1% in the simulation. The horizontal line in each panel represents the theoretical equilibrium value. The initial condition was set to be just one allele (all shared) in each subpopulation. (a) The stochastic pattern for the total-population entropy ^1^ *H*_*T*_ is shown in black curve, and the red curve is ^1^ *H*_*T*_ = *ψ*[1/(1−^2^ *H*_*T*_)]+0.5772, which is the ^1^ *H*_*T*_ value calculated from a function of heterozygosity under IAM-FIM. (b) The pattern for subpopulation entropy ^1^ *H*_*S*_ is shown in black curve, and the red curve is obtained via a link from heterozygosity (see Eq. D7 in S4 Appendix) under IAM-FIM. In both (a) and (b), the processes converge roughly after 40000 generations, but the two lines become close before equilibrium (around 20000 generations). (c) The stochastic pattern for total- population entropy ^1^ *H*_*T*_ under SMM-FIM is shown in black curve, and the red curve is log{[1+^2^ *H*_*T*_−(^2^ *H*_*T*_)^2^]/(1−^2^ *H*_*T*_)}, which is the ^1^ *H*_*T*_ value calculated from a function of heterozygosity. (d) The pattern for subpopulation entropy ^1^ *H*_*S*_ is shown in black curve, and the red curve is obtained via a link from heterozygosity (see S4 Appendix for the link). The relationship between heterozygosity and Shannon entropy exists in all stages of the stochastic process under SMM-FIM.

We used simulations to calculate Shannon entropy in two ways: directly from the simulated allelic data, and predicted from heterozygosity via the equations for FIM, as described in the preceding paragraph and also noted in the figure caption. Representative outputs are presented to show that the simulated curve and the curve predicted from heterozygosity for the total population (Fig 3A) and for a subpopulation (Fig 3B) under IAM-FIM. The corresponding plots for SMM-FIM are shown in Fig 3C and 3D. These simulations results were averaged over 5 loci, which each start out fixed for a single allele. Our simulation results showed that the Shannon entropy curve predicted from the heterozygosity values for IAM-FIM is slightly lower than the simulated line, but the two lines become very close even before equilibrium is reached, revealing that the relationship is also approximately valid before the equilibrium is attained. For SMM-FIM, the simulated curve and the curve predicted from heterozygosity match very closely, and almost overlap starting from the initial stages when the initial population is fixed for a single allele, shared by all subpopulations.

## Empirical Test

Simulations in the preceding section show that our predicted relationship between Shannon entropy and heterozygosity is approximately valid under some non-equilibrium conditions; and for SMM the relationship is valid even in nearly all stages of the process. By examining real populations of various ages, we can test the robustness of these relationships in practice. Starlings were introduced to south-eastern Australia in the mid-19th century [73–76] and provide a good test case, having several populations of different ages. Since the 1970s, starlings have begun to invade Western Australia [77] and have been intensively controlled since that time.

Rollins [74,75] used genetic markers to trace the possible invasion pathways. Using starlings captured in 17 localities throughout their Australian range, four genetically distinct starling subpopulations were identified and their localities are shown in the footnotes of Table A (S5 Appendix), and are numbered 1−4 in order from west to east. Subpopulations 1 and 2 are the youngest, being established approximately 5 and 35 years (respectively) before the time of sampling, while subpopulations 3 and 4 are older, having been established in the 19^th^ century. Since generation time is about three years [74], the subpopulations cannot be in equilibrium or near equilibrium, especially the two youngest populations, 1 and 2.

We consider two types of data, which have different expected models [74]. (1) A locus which is expected to follow the IAM: Dopamine receptor D4 (*DRD4*) allele frequency data for the four subpopulations (Table A of S5 Appendix). (2) Three loci which are expected to follow the SMM: microsatellite data for 3 loci for the four subpopulations (Table B of S5 Appendix) [75]. While we expect these microsatellites to be selectively neutral, there is some evidence of selection on *DRD4* in other avian taxa [78,79]. Rollins [74] explicitly tested the *DRD* data used here for departures from neutrality (Tajima’s *D*, Fu’s *F*) and found no evidence of selection at this locus in the starlings included in our analysis. The graded series of Australian starling populations of different known ages provided us with the possibility of investigating approach to equilibrium, and robustness to non-equilibrium situations [80].

Based on allele frequencies (*DRD4* and microsatellite data), statistical estimation techniques are applied to obtain bias-corrected estimates of heterozygosity, Shannon entropy, Shannon differentiation and other parameters [53,81]; these estimates are referred to as “empirical” (or “estimated”) values in tables and the following discussions. The bias-correction is necessary because parameters/measures based directly on observed frequencies are biased. All the statistical estimation method for calculating the empirical values from sample data is summarized in S4 Appendix. The procedures to obtain the expected values under different models (IAM, SMM, IAM-FIM, and SMM-FIM) are summarized in S4 Appendix, and also briefly described below.

### DRD4 data

Using the *DRD4* data, we did two independent analyses. (a) We performed analysis under IAM by treating each of the four subpopulations as completely isolated from each other; all results are summarized in Table 2 and described below. (b) We treated the four populations as partially-connected subpopulations under IAM-FIM; all results and comparisons are summarized in Table 3.

**Table 2:** Consistency of empirical data with IAM based on the Dopamine receptor D4 (*DRD4*) alleles data.

Method/Model	Measure	Subpopu-	Subpopu-	Subpopu-	Subpopu-
		lation 1	lation 2	lation 3	lation 4
Empirical	Estimated Shannon	**2.0539**	**2.2415**	**2.6845**	**2.7638**
	(s.e.)	(0.0952)	(0.1139)	(0.0460)	(0.0815)
	Estimated heterozygosity	0.8018	0.8688	0.9004	0.8949
	(s.e.)	(0.0232)	(0.0193)	(0.0059)	(0.0147)
IAM expected^#^	Expected Shannon	**2.0933**	**2.5410**	**2.8336**	**2.7763**
	(s.e.)	(0.1250)	(0.1392)	(0.0608)	(0.1426)
	Proportional difference	0.0188	0.1179	0.0526	0.0045

Empirical and expected values by treating each of the four subpopulations as an isolated population following IAM for mutation. Data are shown in Table A (S5 Appendix). See Table 1 for the expected formulas and S4 Appendix for statistical methods to obtain empirical values. The proportional difference PD ≡ (expected value−estimated value)/expected value. All s.e. estimates were obtained by a bootstrap method based on 1000 resamples generated from the observed allele frequency distribution.

^#^The expected parameters under IAM for the four subpopulations: *Nμ* = (1.0113, 1.6552, 2.2610, 2.1280); see Eq 1.

**Table 3:** Consistency of empirical data with IAM-FIM based on the Dopamine receptor D4 (*DRD4*) alleles data.

Method or	Measure	Total	Subpopu-	Shannon	Jost	*G*_*ST*_
assumptions		population	lation	differentiation	differentiation	
Empirical	Estimated Shannon	**2.7444**	**2.4359**	**0.2225**		
	(s.e.)	(0.0400)	(0.0447)	(0.0226)		
	Estimated heterozygosity	0.9106	0.8665		0.4407	0.0485
	(s.e.)	(0.0046)	(0.0083)		(0.0386)	(0.0070)
IAM-FIM						
expected^#^	Expected Shannon	**2.9466** ^*^	**2.3918** ^§^	**0.4002**		
	(s.e.)	(0.0524)	(0.0626)	(0.0315)		
	Proportional difference	0.0686	-0.0184	0.4440		

Empirical and IAM-FIM expected values for total-population, subpopulation and differentiation measures under IAM-FIM. Data are shown in Table A (S5 Appendix). See Table 1 for the expected formulas and S4 Appendix for statistical methods to obtain empirical values. The proportional difference PD ≡ (expected value−estimated value)/expected value. All s.e. estimates were obtained by a bootstrap method based on 1000 resamples generated from the observed allele frequency distribution.

^#^ The expected parameters under IAM-FIM: *Nμ* = 0.6058, *Nm* = 3.0748; see Eqs. D5 and D6 of S4 Appendix.

^*^ Total population entropy value calculated from total population-heterozygosity under IAM via Eq 3A: ^1^ *H*_*T*_ = *ψ*[1/(1−^2^ *H*_*T*_)]+0.5772.

^§^ Subpopulation entropy is calculated from heterozygosity via a link described in Eq. D7 in S4 Appendix.

**(a) Treating each of the four subpopulations as an isolated population following IAM for mutation (Table 2).** The sample sizes for *DRD4* data from subpopulations 1−4 were 146, 52, 486 and 176 respectively, revealing 16, 11, 31 and 25 alleles, a total of 38 different alleles over all subpopulations (S5 Appendix). Table 2 gives the empirical Shannon entropy values along with estimated s.e. (to quantify sampling errors) from subpopulation 1 to subpopulation 4. The empirical Shannon entropies are H^1 = 2.0539 (s.e. 0.0952), 2.2415 (s.e. 0.1139), 2.6845 (s.e. 0.0460), and 2.7638 (s.e. 0.0815) respectively, which shows an increasing pattern from west to east, consistent with the history of invasion. In our analysis, all s.e. estimates were obtained by a bootstrap method based on 1000 resamples generated from the observed allele frequency distribution. Table 2 also gives the empirical heterozygosity values and s.e from subpopulations 1−4, based on unbiased estimation theory (see S4 Appendix).

The IAM expected values in Table 2 are obtained via the relationship (Eq 3A) ^1^ *H* = *ψ*[1/(1−^2^ *H*)]+0.5772 under IAM within each subpopulation using the assumptions of equilibrium and complete isolation. Both of these assumptions are likely to be violated by the starling subpopulations. Nevertheless, our relationship still accurately predicts their observed entropies (except for subpopulation 2 due to relatively low sample size). The proportional differences (PD) between observed and predicted entropy values for subpopulations 1−4 are respectively 1.88%, 11.79%, 5.26% and 0.45%. Except for subpopulation 2 (in which sample size is relatively low and thus the s.e. of H^1 is relatively high), this relationship therefore appears to be robust for IAM loci in real populations, even if they are far from equilibrium and even if they are not completely isolated. Here the bootstrap method can take into account model uncertainty in the estimation procedures. Thus the uncertainty in estimating heterozygosity was incorporated in our estimated error of the expected Shannon entropies. Note that the s.e. for the expected Shannon entropy (via estimated heterozygosity under FIM assumptions and under equilibrium status) in each case is higher than s.e. of the estimated Shannon entropy (based on data only) due to the propagation effect of model uncertainty on the expected Shannon entropies. This is also valid in nearly all cases in the following discussions.

**(b) Assuming IAM-FIM for the four subpopulations (Table 3).** Under IAM-FIM, Table 3 first gives the empirical results of Shannon entropy and heterozygosity for the total population, subpopulation (the mean of the empirical subpopulation Shannon entropies) and three related differentiation measures: Shannon’s differentiation, Jost’s *D* and *G*_*ST*_. See S4 Appendix for details. The difference between the empirical Shannon entropy for the total population (2.7444) and subpopulation (2.4359) is the empirical mutual information. Thus, it follows from Eq 10 that the estimated Shannon differentiation is (2.7444–2.4359) / log4 = 0.2225. Based on the empirical heterozygosities, Jost’s differentiation measure *D* is estimated to be 0.4407, while *G*_*ST*_ is much lower (0.0485).

For the IAM-FIM expected values, the link between heterozygosity and Shannon entropy for an isolated population (Eq 3A) can also be applied to the total population. This gives an expected total-population entropy of 2.9466, with PD of 6.86% when it is compared with the empirical total-population entropy. The expected subpopulation entropy was computed from the total and subpopulation heterozygosities; see Eq. D7 of S4 Appendix for details. Although the model may be wrong and the equilibrium is unlikely to have been attained, the total-population and subpopulation Shannon entropies are still predicted from heterozygosities with very high relative accuracy (PD = 6.86% for the total-population entropy and—1.84% for subpopulation). However, due to over-prediction for ^1^ *H*_*T*_ (positive PD) and under-prediction for ^1^ *H*_*S*_ (negative PD), the Shannon differentiation calculated is subjected to relatively large PD (44.4%) for these data. The large PD could derive from various departures, from the model, such as selection (although there is no evidence of differential fitness of the *DRD4* genotypes [74]) and the discrepancy between heterozygosity and Shannon entropy (see Fig 3A and 3B), or from stochasticity, heightened by the availability of only a single IAM locus, which is discussed further below, in comparison to the SMM results.

### Microsatellite data

As with the *DRD4* data, we performed two independent analyses based on the allele frequencies for the three microsatellite loci (Locus Sta213, Locus Sta294, and Locus Sta308). (a) We first estimated parameters under SMM separately for each locus by treating each of the four subpopulations as isolated from each other. (b) We treated the four populations as partially-connected subpopulations under under SMM-FIM separately for each locus for the four divided subpopulations. The average results for the three loci for the two studies are shown respectively in Tables 4 and 5. (The results for each locus are provided in S5 Appendix.)

**Table 4:** Consistency of empirical data with SMM based on the microsatellites for each subpopulation (all results are averaged over 3 loci).

Method/Model	Measure	Subpopu-	Subpopu-	Subpopu-	Subpopu-
		lation 1	lation 2	lation 3	lation 4
Empirical	Estimated Shannon	**1.6115**	**1.7696**	**2.0344**	**2.1313**
	(s.e.)	(0.0227)	(0.0484)	(0.0142)	(0.0215)
	Estimated heterozygosity	0.7585	0.7905	0.8491	0.8569
	(s.e.)	(0.0073)	(0.0160)	(0.0034)	(0.0045)
SMM expected^#^	Expected Shannon	**1.6220**	**1.7398**	**2.0272**	**2.1088**
	(s.e.)	(0.0436)	(0.1061)	(0.0313)	(0.0484)
	Proportional difference	0.0065	-0.0171	-0.0036	-0.0107

Empirical and expected values by treating each of the four subpopulations as an isolated population following SMM for mutation. Data are shown in Table B (S5 Appendix). See Table 1 for the expected formulas and S4 Appendix for statistical methods to obtain empirical values. The proportional difference PD ≡ (expected value−estimated value)/expected value. All s.e. estimates were obtained by a bootstrap method based on 1000 resamples generated from the observed allele frequency distribution.

^#^The expected parameters (average over 3 loci) for the four subpopulations: *Nμ* = (2.7901, 3.4202, 6.0214, 8.0434); see Eq 4B.

**Table 5:** Consistency of empirical data with SMM-FIM based on the microsatellites for each subpopulation (all results are averaged over 3 loci).

Methods or	Measure	Total	Subpopu-	Shannon	Jost	*G_ST_*
assumptions		population	lation	differentiation	differentiation	
Empirical	Estimated Shannon	**2.0948**	**1.8867**	**0.1501**		
	(s.e.)	(0.0122)	(0.0153)	(0.0086)		
	Estimated heterozygosity	0.8554	0.8138		0.2983	0.0512
	(s.e.)	(0.0028)	(0.0047)		(0.0185)	(0.0045)
SMM-FIM						
expected^#^	Expected Shannon	**2.0707** ^*^	**1.8773** ^§^	**0.1395**		
	(s.e.)	(0.0166)	(0.0187)	(0.0104)		
	Proportional Difference	-0.0117	-0.0050	-0.0762		

Empirical and SMM-FIM expected values for total-population, subpopulation and differentiation measures under SMM-FIM. Data are shown in Table B (S5 Appendix). See Table 1 for the expected formulas and S4 Appendix for statistical methods to obtain empirical values. The proportional difference PD ≡ (expected value−estimated value)/expected value. All s.e. estimates were obtained by a bootstrap method based on 1000 resamples generated from the observed allele frequency distribution.

^#^ The expected parameters (average over 3 loci) under SMM-FIM: *Nμ* = 6.31, *Nm* = 9.11; see Eqs. D8 and D9 of S4 Appendix.

^*^ Total population entropy value calculated from total population heterozygosity under SMM via Eq 5B of the main text: ^1^ *H*_*T*_≈log{[1+^2^ *H*_*T*_−(^2^ *H*_*T*_)^2^]/(1−^2^ *H*_*T*_)}.

^§^ Subpopulation entropy is calculated from heterozygosity via a link described in S4 Appendix.

**(a) Treating each of the four subpopulations as an isolated population following SMM for mutation (Table 4).** For the three microsatellite loci (Locus Sta213, Locus Sta294, and Locus Sta308), the sample sizes for subpopulations 1–4 are 296, 76, 620 and 274 respectively (except that the sample size for Locus Sta294 in subpopulation 3 is 616). The average numbers of alleles for the four subpopulations are respectively 8.33 (average of 9, 6, 10 for the three loci), 7.66 (9, 7, 7), 10.33 (13, 7, 11) and 11.67 (14, 7, 14); see S5 Appendix for data details. The empirical values tabulated in Tables 4 and 5 were obtained by applying the same methods described for *DRD4* data; see S4 Appendix for formulas.

Table 4 shows that the average of the empirical Shannon entropy values (over 3 loci) from subpopulation 1–4 are respectively H^1 = 1.6115 (s.e. 0.0227), 1.7696 (s.e. 0.0484), 2.0344 (s.e. 0.0142), 2.1313 (s.e. 0.0215), revealing the expected increase with subpopulation age from west to east. Again, the s.e. of the estimated Shannon entropy in subpopulation 2 is higher than those in the other three areas due to relatively low sample size in subpopulation 2. The corresponding empirical heterozygosity values from subpopulations 1–4 also exhibit an increasing trend from west to east, as expected from invasion history.

If these microsatellites follow the single-phase isolated SMM within each subpopulation, Shannon entropy should be related to heterozygosity through the equation (Eq 5B): ^1^ *H* ≈ log{[1+^2^ *H*−(^2^ *H*)^2^]/(1−^2^ *H*)}. Table 4 shows that the average PD values (over 3 loci) between the entropies predicted from heterozygosity and the empirical entropies are 0.65%, −1.71%, −0.36% and −1.07%. The predicted values match the empirical values very closely, even for the youngest populations. Thus, as we have demonstrated in Fig 3C and 3D, for loci that obey single-phase SMM, the relationship between Shannon entropy and heterozygosity applies even to non-equilibrium populations and even if they are not completely isolated, in agreement with our simulation results.

**(b) Assuming SMM-FIM for the four subpopulations (Table 5).** Table 5 gives the average of the empirical results for the total population, subpopulation and three differentiation measures, based on the same methods described for Table 3. As in *DRD4* data, the empirical *G*_*ST*_ (0.0512) is much lower than the Shannon’s differentiation value (0.1501) and Jost’s *D* (0.2983). Applying the same link between heterozygosity and entropy for an isolated population to the total population, we obtain the SMM-FIM expected value of 2.0707 for the total-population entropy, which is very close to the empirical value of 2.0948 (PD = -1.17%). The mean within-subpopulation entropy is also very accurately predicted (PD = -0.50%) from our SMM-FIM theory given in S4 Appendix, even though nearly all the assumptions of the FIM model may not be satisfied, as in this starling population (which is far from equilibrium, with unequal subpopulation sizes, variable number of subpopulations through time, and spatially non-homogeneous migration). The expected Shannon differentiation value is 0.1395, which agrees well with the empirical value of 0.1501 with PD = -7.62%. This good performance compared to the Shannon differentiation for IAM (Table 3, with PD -44.4%) may be simply due to the averaging over three loci in the SMM case (Table 5). This can be seen by the improvement in performance relative to cases where each is analysed separately. The results for each locus are shown in S5 Appendix (Tables C-E), where the PDs for Shannon differentiation are -18.5%, 16.8% and -15.4% for the three loci. We also note that, according to our simulations, the link between heterozygosity and Shannon entropy under SMM-FIM (Fig 3C and 3D) is very robust and valid in nearly all stages. The link applies even in populations that violate two conditions: being far from equilibrium, and being connected by some dispersal.

## Conclusions and Discussion

Geneticists have long known that in an isolated population at equilibrium, the heterozygosity at a neutral locus in equilibrium under IAM is a simple function of the fundamental biodiversity parameter *θ* (= 4*Nμ*). Here we have shown that for neutral alleles in equilibrium, Shannon entropy is also a simple function of *θ* (see Eqs 2A and 2B). It follows that Shannon entropy is also a simple function of heterozygosity (Eq 3A). This provides a novel test for neutrality: if the observed entropy is significantly different from the entropy predicted on the basis of the observed heterozygosity, then the locus violates the assumptions of the neutral model or the IAM mutation model. We have also shown in an isolated population at equilibrium under a single-phase SMM that Shannon entropy is a simple function of *θ* (Eq 5A), and a simple link between heterozygosity and Shannon entropy also exists (Eq 5B). Then a similar test for neutrality for SMM is also provided. All theory for IAM and SMM is valid not only for isolated population but also for the total population under FIM by replacing *θ* with *θ*_*T*_ (= 4*N*_*T*_ *μ*) where *N*_*T*_ denotes the effective size of the total population a finite island model; see Table 1 for a summary.

In Fig 3, we have demonstrated for partially-connected subpopulations under IAM-FIM and SMM-FIM that our new link between entropy and heterozygosity turns out to be quite robust for neutral alleles and is satisfied even before equilibrium is attained, at least when the initial population has low diversity (as is often the case after a founding event). Our simulations and empirical data from starlings introduced to Australia both suggest the robustness of our proposed links.

In Table 1, we summarized all formulas derived in this paper for two mutation models: IAM and SMM. In this paper, we have provided a bridge between the two models. As shown in Table 1, when the parameter *α* in SMM tends to 0 for an isolated population, all formulas reduce to those for IAM. For total population, when *α*_*T*_ in SMM-FIM tend to 0, all formulas for SMM reduce to those for IAM-FIM. For subpopulation, when both *α*_*T*_ and *α*_*S*_ tend to 0, all formulas for SMM-FIM reduce to those for IAM-FIM. Generally, all properties of these two mutation models based on allele proportion distributions can be connected by this bridge.

We are also now able to link Shannon differentiation (normalized mutual information) to the parameters of the finite island model at equilibrium under both IAM-FIM and SMM-FIM. Shannon differentiation, like Jost’s *D*, is zero when all allele distributions are identical in each subpopulation, and is unity when the subpopulations share no alleles. Figs 1 and 2 reveal that Shannon’s differentiation is increasing with mutation rate, and decreasing with dispersal rate if all other parameters are fixed. In Table B (S2 Appendix), we tabulate the expected values of *G*_*ST*_, Jost’s *D* and some simplified formulas for the mutual information under IAM with equilibrium in the FIM. The expected values of *G*_*ST*_ is determined by the sum of dispersal and mutation, *N*(*m* ^*^+*μ*), whereas the expected values of Jost’s *D* is determined by the scaled immigration rate [58,72], a ratio between pairwise dispersal rate and mutation rate, as expressed by the factor *m* ^*^/(*nμ*) = *m*/[(*n*–1)*μ*]. When 4*Nm* ^*^ >> 4*Nnμ* >> 0 or 4*Nnμ*>> 4*Nm* ^*^, Shannon differentiation simplifies greatly, revealing the factors that control it. In the latter case, Shannon differentiation is nearly controlled by the ratio *m* ^*^/(*nμ*), like Jost’s *D*; see the last formula in Table B of S2 Appendix. In the former case, Shannon differentiation is determined by a combination of 4*Nnμ*, *G*_*ST*_ and *D*, or equivalently, by a combination of *N*(*m* ^*^+*μ*) and *m* ^*^/(*nμ*). However, the dependence on *N*(*m* ^*^+*μ*) is very weak when 4*Nnμ* >> 2, and thus the main thing that controls entropy differentiation is the ratio *m* ^*^/(*nμ*); see S2 Appendix for details.

In statistics, information theory, ecology, and physics, Shannon entropy has been generalized into numerous parametric families of “generalized entropies”, which vary in the weight they give to common versus rare alleles (or their analogs in other disciplines). The Tsallis or HCDT generalized entropies of order *q*, and the Rényi entropies [82], are two widely used families. Each family of generalized entropies generates a smooth curve when plotted as a function of the order parameter *q*. When *q* = 0 the generalized entropy ignores allele frequencies (it is a function only of allele number). As *q* increases, the generalized entropies are increasingly sensitive to allele frequencies. At *q* = 1 we have Shannon entropy which weighs alleles according to their population share. Moving beyond *q* = 1, the entropies increasingly emphasize the most abundant alleles. When *q* = 2 the measures use the same allele weighting as heterozygosity. This graph of generalized entropy as a function of *q* is called an “entropy spectrum”, and there is a corresponding “diversity profile” when the entropies are converted to effective number of alleles before plotting them. Either one of these curves completely characterizes a given allele proportion distribution, and carries the same information as the Ewens’ probability density function [55]. In S1 Appendix, we have provided the theoretical expressions for these entropy spectra or diversity profiles in terms of model parameters, under IAM and SMM. This provides a new way of characterizing the neutral equilibrium allele proportion distribution.

Fig 3 shows that it takes tens of thousands of generations to reach equilibrium in the scenarios we considered. However, the starling data (Tables 2–5) show that the methods appear to be quite robust to all but the most extreme deviations from equilibrium in the newest western populations (Subpopulation 2 with relatively sparse data), although even the oldest of the populations was established only of the order of a hundred generations ago. It is encouraging there is generally good fit to real biological data, even with only small numbers of loci, and various known deviations from the theoretical model listed above, although there is better fit when there is averaging over more than one locus, and more time allowed for equilibration (Tables 2–5).

We summarize the major comparisons between the measures based on the traditional heterozygosity and our proposed measures based on Shannon entropy below. This summary also reveals the limitations of each approach.

1. As discussed, heterozygosity and Shannon entropy each contain useful but partial information about an allele frequency distribution. These two measures, along with allele numbers [64,83, p. 263], are the three most informative special cases of a complete profile of the Tsallis entropies or the Rényi entropies. Measures based on the traditional heterozygosity disproportionately favor the frequent alleles whereas measures based on Shannon entropy weigh alleles in proportion to their frequencies.
2. Both the heterozygosity and Shannon entropy and their differentiation measures can be linked to neutral genetic models under equilibrium, e.g., IAM and SMM for an isolated population, and IAM-FIM and SMM-FIM for subdivided populations. These formulas are shown in Table 1. Our formulas based on Shannon entropy in Table 1 for an isolated population are at least as simple as those based on heterozygosity. Although our formulas for subdivided populations and mutual information look more complicated, all can be numerically evaluated using standard software.
3. Under IAM-FIM, the measures *G*_*ST*_ and Jost’s *D*, or equivalently ^2^ *H*_*T*_ and ^2^ *H*_*S*_, can be jointly used to obtain analytic estimates of dispersal rate and mutation rate based on estimated heterozygosities (see Eqs. D5 and D6 in S4 Appendix for the estimation formulas and the footnotes of Table 3 for their estimates as applied to the starling data). Under SMM-FIM, numerical method is required to obtain estimates of dispersal rate and mutation rate (see Eqs. D8 and D9 in S4 Appendix and the footnotes of Table 5 for their estimates as applied to the starling data). However, for measures based on Shannon entropy, currently it is not feasible to obtain analytic or numerical estimates of dispersal rate and mutation rate unless empirical equations are adopted [12,13].
4. The Shannon differentiation measure *C*_1*n*_ based on the between-group component of entropy, obeys stronger monotonicity properties than the *G*_*ST*_ and Jost’s *D* based on the between-group component of heterozygosity. A monotonicity property in Jost et al. [70] implies that Shannon’s differentiation measure always increases any time a new allele is added to any subpopulation, with any abundance, whereas *G*_*ST*_ and Jost’s *D* do not satisfy this property. In S6 Appendix, we further prove that if some copies of an allele that is shared among subpopulations are replaced by copies of unshared alleles, Shannon differentiation measure always increases. We also give a counter-example to show that *G*_*ST*_ and Jost’s *D* do not satisfy this requirement. These monotonicity properties reveal that the Shannon differentiation measure has some good properties that are lacking for measures based on heterozygosity, and these properties may better capture the meaning of differentiation in many contexts, including conservation.
5. The measure *G*_*ST*_ in FIM converges very quickly in the genetic stochastic processes whereas the normalized mutual information based on Shannon entropy converges relatively slowly. This is expected because the maximum possible value of *G*_*ST*_ is constrained by the subpopulation heterozygosity and thus takes values in a very narrow range, whereas the value of the normalized mutual information is not constrained a priori and thus potentially spans the full range [0, 1] no matter what the value of subpopulation entropy.
6. Estimators of Shannon entropy or heterozygosity should be used, instead of calculating their observed values directly from the sample allele frequencies. From the perspective of statistical inference, measures based on heterozygosity can be accurately estimated from incomplete samples nearly without any bias because these measures focus on the frequent alleles, which always appear in samples. However, it is surprisingly non-trivial to make accurate estimates of population entropy based on small samples; it can be proven that no unbiased estimator exists [84]. Recently Chao et al. developed a low-bias entropy estimator [53]. See S4 Appendix for statistical estimation.

In conclusion, the theoretical advances presented here, combined with the estimation theory [53], should entice geneticists to add Shannon entropy to their genetic toolkit, and to develop connections between the entropy of allele proportion distributions, the entropy of gene sequences, the mutual information between gene regions, and other information-theoretical properties of genes. The R scripts for computing all measures discussed in this paper are available in S7 Appendix with comments.

## Supporting Information

S1 Appendix Derivation of the equilibrium expectation of Shannon entropy under IAM and SMM for an isolated population. (PDF)

S2 Appendix Derivation of the equilibrium expectation of total-population and subpopulation Shannon entropy under IAM-FIM. (PDF)

S3 Appendix Derivation of the equilibrium expectation of total-population and subpopulation Shannon entropy under SMM-FIM. (PDF)

S4 Appendix Details for real data analysis. (PDF)

S5 Appendix Dopamine receptor D4 (*DRD4*) and microsatellite data (3 loci) of four starling populations (Group 1-Group 4). (PDF)

S6 Appendix Two strong monotonicity properties for mutual information and Shannon differentiation measure. (PDF)

S7 Appendix R scripts for computing all measures discussed in this paper. (TXT)
