# Supplementary material for: Expected Shannon Entropy and Shannon Differentiation between Subpopulations for Neutral Genes under the Finite Island Model
Source: PLoS One. 2015 Jun 11;10(6):e0125471. doi: 10.1371/journal.pone.0125471 (PMC4465833; doi:10.1371/journal.pone.0125471)
Supplement: S1 Appendix — (PDF) [file pone.0125471.s001.pdf]

Supporting Information

Expected Shannon entropy and Shannon differentiation between subpopulations for neutral genes under the finite island model

Anne Chao, Lou Jost, T. C. Hsieh, K. H. Ma, William B. Sherwin, and Lee Ann Rollins

S1 Appendix. Derivation of the equilibrium expectation of Shannon entropy under IAM and SMM for an isolated population

An isolated population under IAM

As described in the main text, let N denote the number of diploid individuals in an idealized population, and μ denote the averaged mutation rate per generation. We assume that the population size is sufficiently large so that the distribution of allele proportions is essentially continuous; for non-ideal populations, N is replaced by effective population size. Based on Ewens' sampling formula, Sherwin et al. [1] expanded the logarithm function as a power series and obtained Shannon entropy for an idealized population as the following infinite series:

$$ {}^1H = \theta \sum_{j=1}^{\infty} \left\{ [1-(2N)^{-1}]^{j+\theta} / [j(j+\theta)] \right\}, $$

where $\theta = 4N\mu$. Our first step is to simplify the above infinite series. Note that for any $a > 0$ and $b > 0$, we have

$$ \int_0^1 x^{a-1}(1-x)^{b-1}(\log x)dx = \frac{\Gamma'(a)\Gamma(b)}{\Gamma(a+b)} - \frac{\Gamma(a)\Gamma(b)\Gamma'(a+b)}{[\Gamma(a+b)]^2}, \qquad \text{(A1)} $$

where $\Gamma(x) = \int_0^{\infty} u^{x-1}e^{-u}du$ is a gamma function, and $\Gamma(1) = 1$. This integration formula is obtained by the definition of a beta function:

$$ B(a,b) \equiv \frac{\Gamma(a)\Gamma(b)}{\Gamma(a+b)} = \int_0^1 x^{a-1}(1-x)^{b-1}dx . $$

Taking the derivative of both sides of the above with respect to a, we obtain Eq. A1. Letting $a = 1$ and $b = \theta$ in Eq. A1, we can express Shannon entropy as

$$ {}^{1}H \approx -\theta\int_0^1 (1-x)^{\theta-1}(\log x)dx = -\Gamma'(1) + \frac{\Gamma'(\theta+1)}{\Gamma(\theta+1)}. \qquad \text{(A2)}$$

We now notice that the right hand side of the above equation can be expressed in terms of the digamma function ψ, which is defined as

$$\psi(z) = \frac{\Gamma'(z)}{\Gamma(z)}.$$

Thus, the expected entropy can be elegantly expressed by

$$ {}^{1}H = \psi(\theta+1) - \psi(1). \qquad \text{(A3)}$$

For the digamma function, when $\theta \geq 2$, an accurate approximation ([2], Chapter 12) is $\psi(\theta+1) \approx \log(\theta + 0.5)$. Also, it is known that $\psi(1) = -\gamma$, where $\gamma = \lim_{k\to\infty}(\sum_{j=1}^{k}\frac{1}{j} - \log k) \approx$ 0.5772 is the famous Euler's constant. We then get our remarkably simple formula for the expected Shannon entropy

$$ {}^{1}H \approx \log(\theta + 0.5) + 0.5772. \qquad \text{(A4)}$$

The generalized entropy of order q, ${}^{q}H = (1 - \sum_i p_i^q)/(q-1)$ based on Ewens' formula $E[\Sigma_i h(p_i)] = \theta\int_0^1 h(p)p^{-1}(1-p)^{\theta-1}dp$ can be expressed as follows:

$$ {}^{q}H = \frac{1}{q-1}[1 - \theta\int_0^1 p^{q-1}(1-p)^{\theta-1}dp] = \frac{1}{q-1}[1 - \theta\, B(q,\theta)], \quad q > 0.$$

This then can be transformed to obtain the effective number of alleles ${}^{q}D$ (diversity of order q):

$$ {}^{q}D = [1-(q-1)({}^{q}H)]^{1/(1-q)} = [\theta\, B(q,\theta)]^{1/(1-q)}, \quad q > 0. \qquad \text{(A5)}$$

For $q = 0$, let A be the allele number. Then ${}^{0}H = A-1$ and ${}^{0}D = A$. Wright [3] and Watterson [4, p. 263] derived the following approximation formula for allele number:

$$ {}^{0}D = \theta\int_{1/(2N)}^{1} p^{-1}(1-p)^{\theta-1}dp \approx \theta\,\{[(1-1/(2N)]^{\theta-1}\log(2N) - \gamma - \psi(\theta)\}$$

$$\approx \theta\,[\log(2N) - \gamma - \psi(\theta)].$$

Therefore, we can plot complete profiles for the diversity and the generalized entropy under IAM.

An isolated population under SMM

We first review Kimura and Ohta [5] for the equilibrium allele proportion distribution under SMM for an isolated population, so that we can extend the theory to multiple populations and provide a bridge between IAM and SMM. Let $\Phi(y)$ be the allele proportion distribution; here $\Phi(y)dy$ represents the expected number of alleles whose frequencies in the population are in the range $(y, y + dy)$. Consider an allele with proportion y in the population. Following the notation of [5], we let y_{-1} and y_1 be the proportions of adjacent alleles, and let $E(y_{-1} + y_1 \mid y)$ be the conditional expected values of the total proportions of the two adjacent alleles given the allele under consideration has frequency y. Let δy be the change of this allele proportion y per generation. As shown in Kimura and Ohta [5], the mean $M_{\delta y}$ and variance $V_{\delta y}$ of the change δy per generation are respectively,

$$M_{\delta y} = -\mu y + \frac{\mu}{2} E(y_{-1} + y_1 \mid y),$$

$$V_{\delta y} = \frac{y(1-y)}{2N}.$$

Then they applied Wright's [6] formula for the steady-state allele proportion distribution by substituting the above mean and variance into Wright's formula:

$$\Phi(y) = \frac{C_1}{V_{\delta y}} \exp\left(2\int_0^1 \frac{M_{\delta y}}{V_{\delta y}} dy \right),$$

where C_1 is a constant satisfies $\int_0^1 y\Phi(y)dy = 1$. To evaluate the integral, Kimura and Ohta assumed that $E(y_{-1} + y_1 \mid y)$ can be approximated by a faction b of the remaining alleles, that is, $E(y_{-1} + y_1 \mid y) \approx b(1-y)$, and thus

$$M_{\delta y} \approx -\mu y + \frac{b\mu}{2}(1-y).$$

Then they obtained the following allele proportion distribution under SMM for an isolated

population:

$$\Phi(y)=\frac{2NC_1}{y(1-y)}\exp\int_0^1(-\frac{4N\mu}{1-y}+\frac{2Nb\mu}{y})dy$$
$$=Ky^{2Nb\mu-1}(1-y)^{4N\mu-1}\equiv Ky^{\alpha-1}(1-y)^{\theta-1}, \quad \text{(A6)}$$

where $\theta=4N\mu$, $\alpha=2Nb\mu$, and $K=1/B(\alpha+1,\theta)$. Note that in IAM, there is no change in $M_{\delta y}$ due to the mutation of the adjacent alleles, implying $b = 0$ and thus $\alpha=2Nb\mu = 0$. Therefore, the distribution in Eq. A6 reduces to that of IAM when α is 0. This provides a bridge between IAM and SMM. That is, the allele proportion distribution can be regarded as a special case of $\alpha = 0$ in the allele proportion distribution of SMM. Kimura and Ohta [5, p. 2762, Eq. 16] obtained the parameter b (or equivalently, α) via the equilibrium heterozygosity formula derived by Ohta and Kimura [7]:

$$\alpha=2N\mu b=\frac{1+4N\mu-(1+8N\mu)^{1/2}}{(1+8N\mu)^{1/2}-1},$$

which can be simplified to

$$\alpha=\frac{(1+8N\mu)^{1/2}-1}{2}=\frac{(1+2\theta)^{1/2}-1}{2}. \quad \text{(A7)}$$

Based on Eq. A6, the equilibrium heterozygosity can be evaluated:

$$^2H=1-\int_0^1 y^2\Phi(y)dy=1-\frac{\alpha+1}{\theta+\alpha+1}. \quad \text{(A8)}$$

Substituting Eqs.A7 into Eq. A8, we can write the heterozygosity as a function of θ:

$$^2H=1-1/(1+2\theta)^{1/2}. \quad \text{(A9)}$$

Conversely, we can also express θ in terms of the heterozygosity:

$$(1+2\theta)^{1/2}=\frac{1}{1-{}^2H} \quad \text{or} \quad \theta=\frac{1}{2}\left[\frac{1}{(1-{}^2H)^2}-1\right]=\frac{{}^2H(2-{}^2H)}{2\,(1-{}^2H)^2}. \quad \text{(A10)}$$

Applying Ewens [8] formula, the generalized entropy of order q based on the allele proportion distribution (Eq. A6) can be evaluated as

$$ {}^{q}H = \frac{1}{q-1}\left(1 - \int_0^1 y^q \Phi(y)dy\right). $$
$$ = \frac{1}{q-1}\left(1 - \frac{1}{B(\alpha+1,\theta)}\int_0^1 p^{q+\alpha-1}(1-p)^{\theta-1}dp\right) $$
$$ = \frac{1}{q-1}\left(1 - \frac{B(\alpha+q,\theta)}{B(\alpha+1,\theta)}\right), \quad q \geq 0, \qquad \text{(A11)} $$

which can be transformed to obtain the diversity of order q:

$$ {}^{q}D = [1-(q-1)({}^{q}H)]^{1/(1-q)} = \left(\frac{B(\alpha+q,\theta)}{B(\alpha+1,\theta)}\right)^{1/(1-q)}, \quad q \geq 0, \qquad \text{(A12)} $$

Based on (A11) and (A12), we can plot complete profiles for the generalized entropy and the diversity under SMM.

The expected Shannon entropy under SMM is expressed as the following integral

$$ {}^{1}H \approx \frac{1}{B(\alpha+1,\theta)}\int_0^1 (-\log p)p^{\alpha}(1-p)^{\theta-1}dp. $$

From Eq. A1, we have the general formula:

$$ \frac{1}{B(a,b)}\int_0^1 x^{a-1}(1-x)^{b-1}(\log x)dx = \psi(a) - \psi(a+b), \qquad \text{(A13)} $$

which implies

$$ {}^{1}H = \psi(\alpha+\theta+1) - \psi(\alpha+1). $$

If $x \geq 2$ so that $\psi(x+1) \approx \log(x+0.5)$, then we obtain the following approximation entropy under SMM (Eq. 5a in the main text),

$$ {}^{1}H \approx \log\frac{\alpha+\theta+0.5}{\alpha+0.5}. $$

It follows from Eq. A7 that

$$ {}^{1}H \approx \log\frac{\alpha+\theta+0.5}{\alpha+0.5} = \log\frac{(2\alpha+1)+2\theta}{2\alpha+1} = \log\frac{(1+2\theta)^{1/2}+2\theta}{(1+2\theta)^{1/2}}. $$

Then substituting Eq. A10 into the above, we obtain (Eq. 5b in the main text)

$$ {}^{1}H \approx \log \frac{1+{}^{2}H-({}^{2}H)^{2}}{1-{}^{2}H}. $$

Table 1 of the main text (with column label "Isolated population" under the model SMM) summarizes the two special cases of q = 1 and 2. Further, Eq. A10 leads to

$$ {}^{2}D = \frac{1}{1-{}^{2}H} = (1+2\theta)^{1/2}, $$

implying

$$ \theta = \frac{({}^{2}D)^{2}-1}{2}. $$

This subsequently gives the diversity of order 1 (Eq. 5c of the main text)

$$ {}^{1}D = \exp({}^{1}H) \approx \frac{\alpha+\theta+0.5}{\alpha+0.5} = \frac{(1+2\theta)^{1/2}+2\theta}{(1+2\theta)^{1/2}} = \frac{{}^{2}D+({}^{2}D)^{2}-1}{{}^{2}D}. $$

References

1. Sherwin WB, Jabot F, Rush R, Rossetto M. Measurement of biological information with applications from genes to landscapes. Mol Ecol. 2006; 15: 2857-2869.
2. Johnson NL, Kotz S, Balakrishnan N. Continuous univariate distributions. New York: Wiley; 1995.
3. Wright S. Evolution and the genetics of populations. Vol. 2. The theory of gene frequencies. Chicago: University of Chicago Press; 1969.
4. Watterson GA. On the number of segregating sites in genetical models without recombination. Theor Pop Biol. 1975; 7:256-276.
5. Kimura M, Ohta T. Distribution of allelic frequencies in a finite population under stepwise production of neutral alleles. Proc Natl Acad Sci. 1975; 72: 2761-2764.
6. Wright S. The distribution of gene frequencies under irreversible mutation. Proc Natl Acad Sci USA. 1938; 24: 253-259.
7. Ohta T, Kimura M. A model of mutation appropriate to estimate the number of electrophoretically detectable alleles in a finite population. Genet Res. 1973; 22: 201-204.
8. Ewens WJ. The sampling theory of selectively neutral alleles. Theor Popul Biol. 1972; 3: 87-112.
