# Supplementary material for: Expected Shannon Entropy and Shannon Differentiation between Subpopulations for Neutral Genes under the Finite Island Model
Source: PLoS One. 2015 Jun 11;10(6):e0125471. doi: 10.1371/journal.pone.0125471 (PMC4465833; doi:10.1371/journal.pone.0125471)
Supplement: S2 Appendix — (PDF) [file pone.0125471.s002.pdf]

Supporting Information

Expected Shannon entropy and Shannon differentiation between subpopulations for neutral genes under the finite island model

Anne Chao, Lou Jost, T. C. Hsieh, K. H. Ma, William B. Sherwin, and Lee Ann Rollins

S2 Appendix. Derivation of the equilibrium expectation of total-population and subpopulation Shannon entropy under IAM-FIM

Subpopulation Shannon entropy

In Wright's finite island model (FIM) [1], there are n subpopulations each with size N, mutation rate μ per generation, and dispersal rate m per generation, so that the alleles of any subpopulation includes a proportion $m/(n-1)$ randomly chosen from each of the other $n-1$ subpopulations. We define $m^* = mn/(n-1)$ as in Latter [2] for notational simplicity.

Barton and Slatkin [3] derived that the conditional distribution for an allele with proportion x in a subpopulation given its allele proportion in the total population y can be expressed as the following function

$$\phi(x|y) = K(1-x)^{4Nm^*(1-y)+4N\mu-1} x^{4Nm^* y-1}.$$

Here $K = 1/B(4Nm^*(1-y)+4N\mu,\ 4Nm^* y+1)$ is a normalizing constant to satisfy $\int x\phi(x|y)\,dx = 1$, i.e.,

$$K = \frac{\Gamma(4Nm^* + 4N\mu + 1)}{\Gamma[4Nm^*(1-y)+4N\mu]\,\Gamma(4Nm^* y+1)}.$$

Thus, the unconditional distribution of the allele proportion x can be obtained by integrating over all possible y values in the total population with distribution function (see Eq. 6 in the main text)

$$\Phi_T(y) = \frac{\theta_T(1-y)^{\theta_T-1}}{y},\quad 0 \le y \le 1,$$

where $\theta_T = 4N_T\mu = 4Nn\mu + \frac{(n-1)\mu}{m^* + \mu}$, and N_T denotes the effective population size of the total population, $N_T = Nn + (n-1)/[4(m^* + \mu)]$. Then the unconditional allele proportion x in a subpopulation is distributed as

$$\begin{aligned}\Phi_S(x) &= \int_0^1 \phi(x|y) y \Phi_T(y) dy \\ &= \int_0^1 K(1-x)^{4Nm^*(1-y)+4N\mu-1} x^{4Nm^* y-1} \theta_T (1-y)^{\theta_T - 1} dy.\end{aligned} \tag{B1}$$

Based on this unconditional distribution, we express Shannon entropy for a subpopulation as follows:

$${}^1H_S = -\int_0^1 x \log x \Phi_S(x) dx = -\int_0^1\int_0^1 K(1-x)^{4Nm^*(1-y)+4N\mu-1} x^{4Nm^* y} \log x dx\, \theta_T (1-y)^{\theta_T - 1} dy.$$

It follows from Eq. A13 of S1 Appendix that the subpopulation entropy formula is

$$\begin{aligned}{}^1H_S &= \int_0^1 \{\psi[4N(m^* + \mu) + 1] - \psi(4Nm^* y + 1)\} \theta_T (1-y)^{\theta_T - 1} dy \\ &= \psi[4N(m^* + \mu) + 1] - \int_0^1 \psi(4Nm^* y + 1) \theta_T (1-y)^{\theta_T - 1} dy.\end{aligned}$$

Several approximation formulas are derived, and they are summarized in Table A at the end of this appendix. The derivations of those formulas will be readily seen in the rest of this appendix.

Some approximation formulas for subpopulation entropy and MI

Since ${}^1H_T = \psi(\theta_T + 1) - \psi(1)$ (see Table 1 in the main text), the mutual information $MI = {}^1H_T - {}^1H_S$ can be written in terms of digamma functions:

$$MI = [\psi(\theta_T + 1) - \psi(1)] - \left\{\psi[4N(m^* + \mu) + 1] - \int_0^1 \psi(4Nm^* y + 1) \theta_T (1-y)^{\theta_T - 1} dy\right\}. \tag{B2}$$

We first approximate the integral involved in the above *MI* formula to simplify the expression. We treat the integral as an expectation $E\ [\psi(4Nm^* Y + 1)]$ where the

expectation is taken with the random variable Y having a density function $f(y)=\theta_T(1-y)^{\theta_T-1}, 0\le y\le 1$, with the following moments:

$$E(Y)=\frac{1}{\theta_T+1},\quad \mathrm{var}(Y)=\frac{\theta_T}{(\theta_T+1)^2(\theta_T+2)},$$

$$E(Y^k)=\frac{k!}{(\theta_T+1)(\theta_T+2)...(\theta_T+k)},\quad k=1,2,\ldots$$

Expanding $\psi(4Nm^*Y+1)$ at the mean $E(Y)=1/(1+\theta_T)$ by a Taylor series and then taking the expectation, we obtain

$$E[\psi(4Nm^*Y+1)]=\psi\left(\frac{4Nm^*}{\theta_T+1}+1\right)+\sum_{k=1}^{\infty}\frac{(4Nm^*)^k}{k!}\psi^{(k)}\left(\frac{4Nm^*}{\theta_T+1}+1\right)E\left(Y-\frac{1}{\theta_T+1}\right)^k, \tag{B3}$$

where $\psi^{(k)}(a)$ denotes the k-th derivative of the digamma function evaluated at the point a. Specifically, here we have $a=[4Nm^*/(\theta_T+1)]+1$. In our applications, $\psi^{(k)}(a)$ is dominated by the first term, i.e., $\psi^{(k)}(a)\approx(-1)^{k+1}(k-1)!a^{-k}+O(a^{-k-1})$; see [4]. Thus the infinite series in Eq. B3 is an alternating series, and the term for k =1 vanishes because $E(Y)$ $=1/(1+\theta_T)$. In most simulation scenarios we conducted, only the term for k = 2 dominates the infinite sum. So we suggest the following approximation formula:

$$E\ [\psi(4Nm^*Y+1]\approx\psi\left(\frac{4Nm^*}{\theta_T+1}+1\right)+\frac{(4Nm^*)^2}{2}\psi''\left(\frac{4Nm^*}{\theta_T+1}+1\right)Var(Y)$$

$$=\psi\left(\frac{4Nm^*}{\theta_T+1}+1\right)-\frac{1}{2}\left(\frac{4Nm^*}{4Nm^*+\theta_T+1}\right)^2\frac{\theta_T}{(\theta_T+2)}.$$

This leads to a general approximation for subpopulation Shannon entropy (displayed in Table A) and also our proposed approximation formula for MI (Table B):

$$MI\approx[\psi(\theta_T+1)-\psi(1)]-\left[\psi[4N(m^*+\mu)+1]-\psi\left(\frac{4Nm^*}{\theta_T+1}+1\right)\right]-\frac{1}{2}\left(\frac{4Nm^*}{4Nm^*+\theta_T+1}\right)^2\frac{\theta_T}{(\theta_T+2)}. \tag{B4}$$

We could obtain a more accurate approximation formula by retaining more terms in the infinite sum in Eq. B3, but our numerical results have suggested that the approximation

formula (Eq. B4) works well except for the special case of two subpopulations (n = 2). Note that in Eq. B4, Nm^* is interpreted as the number of dispersals per generation in a subpopulation and $\theta_T + 1$ is interpreted as the effective number of mutations per generation in the total population. Since $\theta_T + 1 = 4Nn\mu + \frac{m^* + n\mu}{m^* + \mu}$, implying when $m^* >> n\mu$, we have $\theta_T + 1 \approx 4Nn\mu + 1$ and thus $4Nm^*/(\theta_T + 1) \approx m^*/[n\mu + (1/4N)] \approx m^*/n\mu$. We provide below simple approximation formulas under three conditions:

(1) $4Nm^* >> 4Nn\mu >> 0$; (2) $4Nm^* >> 4Nn\mu$ and $4Nn\mu$ is small; (3) $4Nn\mu >> 4Nm^*$.

In each case, we obtain both the approximate subpopulation entropy formulas (summarized in Table A) and the corresponding *MI* formulas (summarized in Table B).

(1) *An approximation formula when* $4Nm^* >> 4Nn\mu >> 0$

We can further simplify our approximation formula (Eq. B4) when $4Nm^* >> 4Nn\mu >> 0$. In this case, each of the three digamma functions (except for $\psi(1)$) can be simply approximated by a logarithm function, i.e.,

$$[\psi(\theta_T + 1) - \psi(1)] - \left[\psi[4N(m^* + \mu) + 1] - \psi\left(\frac{4Nm^*}{\theta_T + 1} + 1 \right) \right]$$

$$\approx \log(\theta_T + 1) - \psi(1) - \log[4N(m^* + \mu) + 1] + \log\left(\frac{4Nm^*}{\theta_T + 1} + 1 \right)$$

$$= \log\left(\frac{1 + \frac{m^*}{n\mu}}{\frac{1}{n} + \frac{m^*}{n\mu}} \right) - \psi(1).$$

Adding the last term of Eq. B4 and substituting $\theta_T = 4Nn\mu/(1 - G_{ST})$, we obtain the following *MI* approximation for $4Nm^* >> 4Nn\mu >> 0$:

$$MI \approx \log\left(\frac{1 + \frac{m^*}{n\mu}}{\frac{1}{n} + \frac{m^*}{n\mu}} \right) - \psi(1) - \frac{1}{2}\left(1 + \frac{n\mu}{m^*}\right)^{-2} \left(\frac{4Nn\mu}{4Nn\mu + 2(1 - G_{ST})} \right)$$

$$= -\log\left(1 - D + \frac{D}{n}\right) - \psi(1) - \frac{1}{2}(1 - D)^2 \left(\frac{4Nn\mu}{4Nn\mu + 2(1 - G_{ST})} \right). \qquad \text{(B5)}$$

The corresponding approximation for subpopulation entropy is given in Table A. Here $D = 1/[1 + m^* /(n\mu)]$ denotes Jost's differentiation measure *D* [5, Eq. 17]. The above approximation shows that when $4Nm^* >> 4Nn\mu >> 0$, *MI* is approximated by a simple function of G_{ST} and Jost's *D*. Since G_{ST} is determined by the sum of dispersals and mutation, $4N(m^* + \mu)$, whereas the expected values of Jost's *D* is determined by the ratio $m^* /(n\mu)$ (Table B), Eq. B5 implies that Shannon differentiation is controlled by a combination of $N(m^* + \mu)$ and $m^* /(n\mu)$. When $4Nn\mu >> 2$, then we have $4Nn\mu + 2(1 - G_{ST}) \approx 4Nn\mu$, and thus *MI* is a function of the ratio $m^* /(n\mu)$ only. Also, the dependence on $N(m^* + \mu)$ is very weak when $4Nn\mu >> 2$. In this case, the main factor that controls Shannon differentiation is the ratio $m^* /(n\mu)$.

(2) *An approximation formula when* $4Nm^* >> 4Nn\mu$ and $4Nn\mu$ is small

The condition $4Nm^* >> 4Nn\mu$ implies that the second term in $\theta_T = 4Nn\mu + \dfrac{(n-1)\mu}{m^* + \mu}$ can be ignored and thus $\theta_T \approx 4Nn\mu$. Then, when $4Nn\mu$ is small, we have the following two expansions at $\theta_T = 0$ to the second-order terms:

$$\psi(\theta_T + 1) \approx \psi(1) + [\psi'(1)]\theta_T + \frac{1}{2}[\psi''(1)]\theta_T^2,$$

$$\psi\left(\frac{4Nm^*}{\theta_T + 1} + 1\right) \approx \psi(4Nm^* + 1) - (4Nm^*)[\psi'(4Nm^* + 1)]\,\theta_T$$

$$+ (4Nm^*)[\psi'(4Nm^* + 1)]\,\theta_T^2 + \frac{1}{2}(4Nm^*)^2[\psi''(4Nm^* + 1)]\,\theta_T^2.$$

Since $\psi'(1) = 1.6449$, $\psi''(1) = -2.4041$, $\psi'(4Nm^* + 1) \approx (4Nm^* + 1)^{-1}$ and $\psi''(4Nm^* + 1) \approx -(4Nm^* + 1)^{-2}$, the two expansions given above and Eq. B4 lead to the following approximation of *MI*:

$$MI \approx \left(1.6449 - \frac{4Nm^*}{4Nm^* + 1}\right)\theta_T + \left(-1.2021 + \frac{4Nm^*}{4Nm^* + 1} - \frac{1}{2}\frac{(4Nm^*)^2}{(4Nm^* + 1)^2}\right)\theta_T^2$$

$$-\frac{1}{2}\left(\frac{4Nm^*}{4Nm^*+\theta_T+1}\right)^2\frac{\theta_T}{(\theta_T+2)}.$$

If we can further assume that $4Nm^*$ is sufficiently large so that $4Nm^*/(4Nm^*+1)\approx 1$, then the above can be simplified to a simple function of $4Nn\mu$ and D:

$$MI\approx 0.6449(4Nn\mu)-0.7021(4Nn\mu)^2\ -\frac{1}{2}(1-D)^2\left(\frac{4Nn\mu}{4Nn\mu+2}\right). \tag{B6}$$

In the extreme case in which $4Nm^* >> 4Nn\mu\to 0$, *MI* approaches 0 and thus Shannon differentiation $1-C_{1n}$ approaches 0. In the derivation, it is readily seen that an approximation of subpopulation Shannon entropy (given in Table A) is

$$^1H_S\approx(4Nn\mu)-\frac{1}{2}(4Nn\mu)^2\ +\frac{1}{2}\left(1+\frac{n\mu}{m^*}\right)^{-2}\left(\frac{4Nn\mu}{4Nn\mu+2}\right).$$

(3) ***An approximation formula when*** $4Nn\mu >> 4Nm^*$

The assumption $4Nn\mu >> 4Nm^*$ implies $E(Y)$ is small. Thus we can expand the integrand $\psi(4Nm^*Y+1)$ in Eq. B2 at $Y=0$ and take expectation with the random variable Y having a density function $f(y)=\theta_T(1-y)^{\theta_T-1}$, $0\le y\le 1$, as in the derivation of Eq. B3. Then

$$E[\psi(4Nm^*Y+1)]\approx\psi(1)+\sum_{k=1}^{\infty}\frac{(4Nm^*)^k}{k!}\psi^{(k)}(1)[E(Y^k)].$$

where $\psi^{(k)}(1)$ denotes the derivative of the digamma function evaluated at the point 1. Substituting the moments $E(Y^k)$, we obtain

$$E\ [\psi(4Nm^*Y+1)]\approx\psi(1)+\sum_{k=1}^{\infty}[\psi^{(k)}(1)]\frac{(4Nm^*)^k}{(\theta_T+1)(\theta_T+2)...(\theta_T+k)}.$$

Thus *MI* can be expressed as

$$MI=\{\psi(\theta_T+1)-\psi[4N(m^*+\mu)+1]\}+\sum_{k=1}^{\infty}[\psi^{(k)}(1)]\frac{(4Nm^*)^k}{(\theta_T+1)(\theta_T+2)...(\theta_T+k)}.$$

If we retain the first two terms in the infinite series and substitute $\psi'(1)$=1.6449, $\psi''(1)=$ −2.4041 into the above formula, then the following approximation formula is obtained:

$$MI \approx \psi(\theta_T + 1) - \psi[4N(m^* + \mu) + 1] + 1.6449\left(\frac{4Nm^*}{\theta_T + 1}\right) - 2.4041\left(\frac{4Nm^*}{\theta_T + 1}\right)\left(\frac{4Nm^*}{\theta_T + 2}\right).$$

Since $\theta_T + 1 = 4Nn\mu + \frac{m^* + n\mu}{m^* + \mu}$, and $1 \le \frac{m^* + n\mu}{m^* + \mu} \le n$, implying when $4Nn\mu >> n$, we have $\theta_T + 1 \approx 4Nn\mu$ and $4Nm^* / (\theta_T + 1) \approx m^* / (n\mu)$. Then the two digamma functions can be approximated by logarithm functions, leading to

$$MI \approx \log \frac{4Nn\mu}{4N(m^* + \mu)} + 1.6449\left(\frac{m^*}{n\mu}\right) - 2.4041\left(\frac{m^*}{n\mu}\right)^2.$$

$$\approx -\log\left(\frac{1}{n} + \frac{m^*}{n\mu}\right) + 1.6449\left(\frac{m^*}{n\mu}\right) - 2.4041\left(\frac{m^*}{n\mu}\right)^2. \qquad \text{(B7)}$$

When $m^* / (n\mu)$ approaches 0, we have $MI \to \log n$ and Shannon differentiation $1 - C_{1n} \to 1$, as expected. A by-product of the above derivation is an approximation formula for subpopulation Shannon entropy: (shown in Table A)

$${}^1H_S \approx \psi[4N(m^* + \mu) + 1] - \psi(1) - 1.6449\left(\frac{m^*}{n\mu}\right) + 2.4041\left(\frac{m^*}{n\mu}\right)^2.$$

In the extreme case $m^* \to 0$ and $m^* / (n\mu) \to 0$, which means each subpopulation is an isolated subpopulation, then the above subpopulation entropy tends to $\psi(4N\mu + 1) - \psi(1)$, which is our formula for an isolated population (see Table 1 of the main text).

We summarize the above findings below and in Table B:

(1) When $4Nm^* >> 4Nn\mu >> 0$, *MI* is approximated by a simple function of $4Nn\mu$, G_{ST} (and Jost's *D* (Eq. B5), revealing both $4N(m^* + \mu)$ (main factor which determines G_{ST}) and $m^* / (n\mu)$ (main factor which determines Jost's *D*) affect Shannon differentiation. If the number of mutations is large enough (i.e., $4Nn\mu >> 2$), the ratio $m^* / (n\mu)$ becomes the dominating factor.

(2) In the extreme case in which $4Nm^* >> 4Nn\mu$ and $4Nn\mu$ is small, *MI* is a simple function of $4Nn\mu$ and Jost's *D* (Eq. B6). In the extreme case that $4Nn\mu \to 0$, both *MI* and

Shannon differentiation measures approach the minimum value of 0.

(3) In the opposite extreme case in which $4Nn\mu >> 4Nm^* \to 0$, MI is a simple function of $m^*/(n\mu)$ (Eq. B7). When $m^*/(n\mu)$ approaches 0, we have $MI \to \log n$ and Shannon differentiation approaches the maximum value of unity.

The generalized entropy of order q for subpopulations

Although the subpopulation heterozygosity is well known in the literature [6,7], here we derive it directly from the following allele proportion distribution for a subpopulation (Eq. B1):

$$\Phi_S(x) = \int_0^1 K(1-x)^{4Nm^*(1-y)+4N\mu-1} x^{4Nm^*y-1} \theta_T (1-y)^{\theta_T-1} dy .$$

The formula can be seen from the following:

$$^2H_S = 1 - \int_0^1 x^2 \Phi_S(x) dx$$

$$= 1 - \int_0^1\int_0^1 K(1-x)^{4Nm^*(1-y)+4N\mu-1} x^{4Nm^*y+1} dx\, \theta_T (1-y)^{\theta_T-1} dy$$

$$= 1 - \int_0^1 K \frac{\Gamma[4Nm^*(1-y)+4N\mu]\Gamma(4Nm^*y+2)}{\Gamma[4N(m^*+\mu)+2]} \theta_T (1-y)^{\theta_T-1} dy$$

$$= 1 - \int_0^1 \left[\frac{4Nm^*y+1}{4Nm^*+4N\mu+1} \right] \theta_T (1-y)^{\theta_T-1} dy$$

$$= 1 - \frac{1}{\theta_T+1} \left(\frac{4Nm^*+\theta_T+1}{4Nm^*+4N\mu+1} \right)$$

$$= 1 - \frac{4Nm^*/(\theta_T+1)+1}{4N(m^*+\mu)+1} = 1 - \left(4Nn\mu \frac{m^*+\mu}{m^*+n\mu} + 1 \right)^{-1} .$$

The generalized entropy of order q can be expressed as

$$\frac{1}{q-1} \left(1 - \int_0^1 x^q \Phi_S(x) dx \right),$$

where

$$\int_0^1 x^q \Phi_S(x)dx$$

$$= \int_0^1 \int_0^1 K(1-x)^{4Nm^*(1-y)+4N\mu-1} x^{4Nm^* y+q-1} dx\, \theta_T (1-y)^{\theta_T -1} dy$$

$$= \int_0^1 K \frac{\Gamma[4Nm^*(1-y)+4N\mu]\Gamma(4Nm^* y+q)}{\Gamma[4N(m^*+\mu)+q]} \theta_T (1-y)^{\theta_T -1} dy$$

$$= \int_0^1 \frac{\prod_{k=1}^{q-1}(4Nm^* y+k)}{\prod_{k=1}^{q-1}[4N(m^*+\mu)+k]} \theta_T (1-y)^{\theta_T -1} dy \ . \qquad \text{(B8)}$$

Although the formula looks complicated, it can be numerically evaluated using standard software.

Table A. The expected subpopulation Shannon entropy under IAM with equilibrium in the FIM. $m^* = mn/(n-1)$, $\theta_T = 4Nn\mu + (n-1)\mu/(m^* + \mu)$.

Conditions	Expected formula for subpopulation Shannon entropy
Exact formula	$^1H_S = \psi[4N(m^* + \mu) + 1] - \int_0^1 \psi(4Nm^* y + 1)\theta_T (1-y)^{\theta_T - 1} dy$ (Eq. 7c of the main text)
General approximation formula	$^1H_S \approx \left[\psi[4N(m^* + \mu) + 1] - \psi\left(\frac{4Nm^*}{\theta_T + 1} + 1\right)\right] + \frac{1}{2}\left(\frac{4Nm^*}{4Nm^* + \theta_T + 1}\right)^2 \frac{\theta_T}{(\theta_T + 2)}$ (Eq. 7d in the main text)
$4Nm^* >> 4Nn\mu >> 0$	$^1H_S \approx \log\left(\frac{4N(m^* + \mu) + 1}{\frac{m^*}{n\mu} + 1}\right) + \frac{1}{2}\left(1 + \frac{n\mu}{m^*}\right)^{-2}\left(\frac{4Nn\mu}{4Nn\mu + 2(1 - G_{ST})}\right)$
$4Nm^* >> 4Nn\mu$, $4Nn\mu$ is small	$^1H_S \approx (4Nn\mu) - \frac{1}{2}(4Nn\mu)^2 + \frac{1}{2}\left(1 + \frac{n\mu}{m^*}\right)^{-2}\left(\frac{4Nn\mu}{4Nn\mu + 2}\right)$
$4Nn\mu >> 4Nm^*$	$^1H_S \approx \psi[4N(m^* + \mu) + 1] - \psi(1) - 1.6449\left(\frac{m^*}{n\mu}\right) + 2.4041\left(\frac{m^*}{n\mu}\right)^2$

Table B. The expected values of G_{ST}, Jost's *D* and mutual information under IAM with equilibrium in the FIM. $m^* = mn/(n-1)$, $\theta_T = 4Nn\mu + (n-1)\mu/(m^* + \mu)$. The proposed Shannon differentiation is $1 - C_{1n} = MI/(\log n)$, where *MI* denotes mutual information. See S2 Appendix for derivations.

Differentiation measures	Expected formula under IAM equilibrium in the FIM
G_{ST}	$G_{ST} = \dfrac{1}{1 + 4N\dfrac{n}{n-1}(m^* + \mu)}$
Jost's $D = 1 - C_{2n}$	$D = \dfrac{1}{1 + m^*/(n\mu)}$
Mutual information (Exact formula)	$MI = [\psi(\theta_T + 1) - \psi(1)] - \psi[4N(m^* + \mu) + 1] + \int_0^1 \psi(4Nm^* y + 1)\theta_T (1-y)^{\theta_T - 1} dy$ (based on Eq. 7c in the main text)
Mutual information (General approximation formula)	$MI \approx [\psi(\theta_T + 1) - \psi(1)] - \left[\psi[4N(m^* + \mu) + 1] - \psi\left(\dfrac{4Nm^*}{\theta_T + 1} + 1\right)\right] - \dfrac{1}{2}\left(\dfrac{4Nm^*}{4Nm^* + \theta_T + 1}\right)^2 \dfrac{\theta_T}{(\theta_T + 2)}$ (based on Eq. 7d in the main text)
Mutual information ($4Nm^* >> 4Nn\mu >> 0$)	$MI \approx \log\left(\dfrac{1 + \dfrac{m^*}{n\mu}}{\dfrac{1}{n} + \dfrac{m^*}{n\mu}}\right) - \psi(1) - \dfrac{1}{2}\left(1 + \dfrac{n\mu}{m^*}\right)^{-2}\left(\dfrac{4Nn\mu}{4Nn\mu + 2(1 - G_{ST})}\right)$ $= -\log\left(1 - D + \dfrac{D}{n}\right) - \psi(1) - \dfrac{1}{2}(1-D)^2\left(\dfrac{4Nn\mu}{4Nn\mu + 2(1 - G_{ST})}\right)$
Mutual information ($4Nm^* >> 4Nn\mu$, $4Nn\mu$ is small)	$MI \approx 0.6449(4Nn\mu) - 0.7021(4Nn\mu)^2 - \dfrac{1}{2}(1-D)^2\left(\dfrac{4Nn\mu}{4Nn\mu + 2}\right)$ $\to 0$ if $4Nn\mu \to 0$
Mutual information ($4Nn\mu >> 4Nm^*$)	$MI \approx -\log\left(\dfrac{1}{n} + \dfrac{m^*}{n\mu}\right) + 1.6449\left(\dfrac{m^*}{n\mu}\right) - 2.4041\left(\dfrac{m^*}{n\mu}\right)^2$ $\to \log n$ if $m^*/n\mu \to 0$

References

1. Wright S. Evolution in Mendelian populations. Genetics. 1931; 16: 97-159.
2. Latter BDH. The island model of population differentiation: a general solution. Genetics. 1973; 73: 147-157.
3. Barton NH, Slatkin M. A quasi-equilibrium theory of the distribution of rare alleles in a subdivided population. Heredity. 1986; 56: 409-415.
4. Abramowitz M, Stegun IA. (eds) Handbook of Mathematical Functions with Formulas, Graphs, and Mathematical Tables. Washington, D. C: National Bureau of Standards, U.S. Department of Commerce. U. S. Government Printing Office; 1972.
5. Jost L. G_{ST} and its relatives do not measure differentiation. Mol Ecol. 2008; 17: 4015-4026.
6. Maruyama T. Effective number of alleles in a subdivided population. Theor Popul Biol. 1970; 1: 273-306.
7. Rousset F. Equilibrium values of measures of population subdivision for stepwise mutation processes. Genetics. 1996; 142: 1357-1362.
