# Supplementary material for: Expected Shannon Entropy and Shannon Differentiation between Subpopulations for Neutral Genes under the Finite Island Model
Source: PLoS One. 2015 Jun 11;10(6):e0125471. doi: 10.1371/journal.pone.0125471 (PMC4465833; doi:10.1371/journal.pone.0125471)
Supplement: S3 Appendix — (PDF) [file pone.0125471.s003.pdf]

Supporting Information

Expected Shannon entropy and Shannon differentiation between subpopulations for neutral genes under the finite island model

Anne Chao, Lou Jost, T. C. Hsieh, K. H. Ma, William B. Sherwin, and Lee Ann Rollins

S3 Appendix. Derivation of the equilibrium expectation of total-population and subpopulation Shannon entropy under SMM-FIM

Allele proportion distribution in the total population

Under SMM-FIM, let N_T be the effective size of the total population, m be the dispersal (or migration) rate, and n be the number of subpopulations. Following the same derivation as that for an isolated population in S1 Appendix, we obtain the mean and variance of the change of an allele with frequency y per generation are the same as those derived in an isolated population, but population size is replaced by the effective population size in the total population. As in an isolated population, let $E(y_{-1}+y_1 \mid y)$ be the conditional expected values of the total proportions of the two adjacent alleles given the allele in the total population has frequency y. Following Kimura and Ohta [1], we assume $E(y_{-1}+y_1 \mid y) \approx b_T(1-y)$. Then the same procedures as those in an isolated distribution lead to the allele proportion distribution in the total population (i.e., replace N and b in Eq. A6 of S1 Appendix by N_T and b_T)

$$\Phi_T(y) = \frac{y^{2N_T b_T \mu - 1}(1-y)^{4N_T\mu - 1}}{B(\alpha_T + 1, \theta_T)} = \frac{y^{\alpha_T - 1}(1-y)^{\theta_T - 1}}{B(\alpha_T + 1, \theta_T)}, \qquad \text{(C1)}$$

where $\theta_T = 4N_T\mu$, $\alpha_T = 2N_T b_T \mu$. Thus, all results in an isolated SMM are also valid for the total population with total-population parameters (α_T, θ_T). For example, the heterozygosity in the total population can be expressed as ${}^2H_T = 1 - 1/(1+8N_T\mu)^{1/2}$ and θ_T and α_T can be written as functions of heterozygosities (see Eqs A7, A10 in S1 Appendix):

$$\theta_T = [1/(1-{}^2H_T)^2 - 1]/2, \quad \alpha_T = [1/(1-{}^2H_T) - 1]/2. \tag{C2}$$

In Table 1 of the main text, we summarize the heterozygosity and Shannon entropy for the total population (with column label “Total population” under the model SMM). The formulas for the total population have the same form as that in an isolated population, with the parameters (α, θ) being replaced by (α_T, θ_T). Also, if $\alpha_T = 0$, then all formulas reduce to those for the total population under IAM-FIM.

Allele proportion distribution in a subpopulation

Let $\Phi_S(x)dx$ represent the expected number of alleles whose proportions in a subpopulation are in the range $(x, x+dx)$. Let $\phi(x|y)$ be the conditional allele proportion distribution of an allele with proportion x in the subpopulation given its allele proportion y in the total population. Following the derivation of Kimura and Ohta [1], we assume that x_{-1} and x_1 are the proportions of adjacent alleles, and let $E(x_{-1} + x_1 \mid x)$ be the conditional expected values of the total proportions of the two adjacent alleles given the allele under consideration has proportion x. Under the assumption $E(x_{-1} + x_1 \mid x) \approx b_S(1-x)$, the mean of the change δx of an allele with proportion x per generation given its proportion in the total population y becomes

$$M_{\delta x} \approx m^*(y-x) - \mu x + \frac{\mu b_S}{2}(1-x).$$

The corresponding variance is $V_{\delta x} \approx \dfrac{x(1-x)}{2N}$. Applying Wright’s formula [2], we obtain the conditional steady-state distribution for allele proportion x in a subpopulation, given its proportion in the total population y, is

$$\phi(x|y) = K_S x^{4Nm^* y + \alpha_S - 1}(1-x)^{4Nm^*(1-y) + 4N\mu - 1}, \tag{C3}$$

where $\alpha_S = 2Nb_S\mu$ and $K_S = 1/B(4Nm^* y + \alpha_S + 1, 4Nm^*(1-y) + 4N\mu)$. Thus we have the marginal allele proportion distribution in a subpopulation:

$$\Phi_S(x) = \int_0^1 \phi(x|y) y \Phi_T(y) dy$$

$$= \frac{1}{B(\alpha_T + 1, \theta_T)} \int_0^1 K_S x^{4Nm^* y + \alpha_S - 1} (1-x)^{4Nm^*(1-y) + 4N\mu - 1} y^{\alpha_T} (1-y)^{\theta_T - 1} dy.$$

Based on this distribution, the heterozygosity of a subpopulation can be evaluated:

$${}^2H_S = 1 - \int_0^1 \int_0^1 x^2 \phi(x \mid y) y \, \Phi_T(y) dx dy = 1 - \frac{4Nm^*(1 - {}^2H_T) + \alpha_S + 1}{4Nm^* + 4N\mu + \alpha_S + 1}.$$

Then we can express α_S as a function of heterozygosities:

$$\alpha_S = 4Nm^* \frac{({}^2H_T - {}^2H_S)}{{}^2H_S} + 4N\mu \frac{(1 - {}^2H_S)}{{}^2H_S} - 1. \quad \text{(C4)}$$

Also, we obtain Shannon entropy for a subpopulation:

$${}^1H_S = \psi(4Nm^* + 4N\mu + \alpha_S + 1) - \int_0^1 \frac{\psi(4Nm^* y + \alpha_S + 1)}{B(\alpha_T + 1, \theta_T)} y^{\alpha_T} (1-y)^{\theta_T - 1} dy. \quad \text{(C5)}$$

Here if both α_T and α_S tend to 0, then the above Shannon entropy and heterozygosity tend to those in IAM-FIM (Eqs. 7b and 7c in the main text). Using a similar expansion we did in S2 Appendix for obtaining Eq. B4 of S2 Appendix, the subpopulation Shannon entropy can be approximated by:

$${}^1H_S \approx \psi(4Nm^* + 4N\mu + \alpha_S + 1) - \psi\left(\frac{4Nm^*(\alpha_T + 1)}{\alpha_T + \theta_T + 1} + \alpha_S + 1 \right)$$
$$+ \frac{1}{2} \left[\frac{4Nm^*}{4Nm^*(\alpha_T + 1) + (\alpha_S + 1)(\alpha_T + \theta_T + 1)} \right]^2 \frac{\theta_T(\alpha_T + 1)}{\alpha_T + \theta_T + 2}.$$

The generalized entropy of order q in a subpopulation is in terms of a complicated integral, but numerically can be evaluated:

$${}^qH_S = \frac{1}{q-1}\left(1 - \int_0^1 x^q \Phi_S(x) dx\right) = \frac{1}{q-1}\left(1 - \frac{\int_0^1 \int_0^1 x^q \phi(x \mid y) y^{\alpha_T} (1-y)^{\theta_T - 1} dx dy}{B(\alpha_T + 1, \theta_T)}\right).$$

References

1. Kimura M, Ohta T. Distribution of allelic frequencies in a finite population under stepwise production of neutral alleles. Proc Natl Acad Sci. 1975; 72: 2761-2764.
2. Wright S. The distribution of gene frequencies under irreversible mutation. Proc Natl Acad Sci USA. 1938; 24: 253-259.
