# Supplementary material for: Expected Shannon Entropy and Shannon Differentiation between Subpopulations for Neutral Genes under the Finite Island Model
Source: PLoS One. 2015 Jun 11;10(6):e0125471. doi: 10.1371/journal.pone.0125471 (PMC4465833; doi:10.1371/journal.pone.0125471)
Supplement: S4 Appendix — (PDF) [file pone.0125471.s004.pdf]

Supporting Information

Expected Shannon entropy and Shannon differentiation between subpopulations for neutral genes under the finite island model

Anne Chao, Lou Jost, T. C. Hsieh, K. H. Ma, William B. Sherwin, and Lee Ann Rollins

S4 Appendix. Details for real data analysis

Statistical estimation method for obtaining empirical values from sample data

Estimation based on data within each subpopulation

It is well known that the "observed" Shannon entropy or heterozygosity based on sample allele frequencies underestimates and thus bias-correction is needed. In Tables 2–5 of the main text, our "empirical" values refer to those unbiased or bias-corrected estimates based on statistical estimation techniques. Let X_i be the sample frequency of allele i and z be the sample size. The unbiased estimator of the heterozygosity is

$$ {}^2\hat{H} = 1 - \sum_i \frac{X_i(X_i - 1)}{z(z-1)} . $$

Let f_1 denote the number of singletons in the sample, and f_2 denote the number of doubletons in the sample. A nearly unbiased of Shannon entropy ${}^1\hat{H}$ is [1]:

$$ {}^1\hat{H} = \sum_{1 \le X_i \le z-1} \frac{X_i}{z} \left(\sum_{k=X_i}^{z-1} \frac{1}{k} \right) + \frac{f_1}{z}(1-A)^{-z+1} \left\{ -\log(A) - \sum_{r=1}^{z-1} \frac{1}{r}(1-A)^r \right\}, \quad \text{(D1)} $$

where

$$ A = \begin{cases} 2f_2/[(z-1)f_1 + 2f_2] & if\ f_2 > 0 \\ 2/[(z-1)(f_1 - 1) + 2] & if\ f_2 = 0, f_1 \ne 0 \\ 1 & if\ f_2 = f_1 = 0. \end{cases} $$

When A = 1, Eq. D1 reduce to

$$^1\hat{H} = \sum_{1 \le X_i \le z-1} \frac{X_i}{z} \left(\sum_{k=X_i}^{z-1} \frac{1}{k} \right).$$

Estimation based on multiple-population data

Assume that there are n subpopulations and there are A distinct alleles in the total population. Suppose a sample is taken from each of the subpopulations, and for simplicity, the sample taken from subpopulation j is referred to as sample j, j = 1, 2, …, n. In sample j, let X_{ij} be the frequency of the i-th allele and $z_j = \sum_i X_{ij}$ be the size of sample j. Throughout the main text and appendices, we assume that all subpopulations are equally-weighted. In this case, basic statistical theory implies that for n equally-weighted subpopulations, an unbiased estimator of the subpopulation heterozygosity ^2H_S is

$$^2\hat{H}_S = 1 - \frac{1}{n} \sum_{j=1}^{n} \sum_i \frac{X_{ij}(X_{ij}-1)}{z_j(z_j-1)}. \tag{D2}$$

An unbiased estimator for the total-population heterozygosity ^2H_T is

$$^2\hat{H}_T = 1 - \frac{1}{n^2} \sum_{j=1}^{n} \sum_i \frac{X_{ij}(X_{ij}-1)}{z_j(z_j-1)} - \frac{1}{n^2} \sum_{j \ne k} \sum_i \frac{X_{ij} X_{ik}}{z_j z_k}. \tag{D3}$$

Within sample j, Shannon's entropy estimator, $^1\hat{H}_j$, j = 1, 2,…, n based on Eq. D1 can be obtained. Then the empirical subpopulation entropy is obtained as the average of these entropy estimates, i.e.,

$$^1\hat{H}_S = \frac{1}{n} \sum_{j=1}^{n} {}^1\hat{H}_j .$$

Let p_{ij} denote the proportion of allele i in the jth subpopulation. The proportion of allele i in the total population is $\bar{p}_{i+} = \sum_{j=1}^{n} p_{ij} / n$. Shannon entropy in the total population is

$$^1H_T = -\sum_{i=1}^{A} \bar{p}_{i+} \log \bar{p}_{i+} .$$

Following Chao & Shen [2], we can apply the Horvitz-Thompson sampling-theory-based approach [3] to obtain an estimator of total-population entropy if all proportions were known:

$$ {}^1\tilde{H}_T = -\sum_i \frac{\bar{p}_{i+} \log \bar{p}_{i+}}{1-\prod_{j=1}^{n}(1-p_{ij})^{z_j}} I(\sum_{j=1}^{n} X_{ij} > 0), \quad \text{(D4)} $$

where $I(A)$ denotes the indicator function, $I(A) = 1$ if A is true, and $I(A) = 0$ otherwise. In practice, we need to estimate all proportions involved in Eq. D4. When there are undetected allele in samples, statistical theory (e.g., see [2]) implies that we cannot simply substitute $\bar{p}_{i+}$ and p_{ij} in Eq. D3 by their sample fractions $\hat{\bar{p}}_{i+} = (1/n)\sum_{j=1}^{n} X_{ij}/z_j$ and $\hat{p}_{ij} = X_{ij}/z_j$. Instead, Chao & Shen [2] substituted p_{ij} by $\tilde{p}_{ij} = \hat{C}_j \sum_{j=1}^{n} X_{ij}/z_j$, where $\hat{C}_j = 1 - f_{1j}/z_j$ is the sample coverage estimator, and f_{1j} is the number of singletons in sample j. To estimate $\bar{p}_{i+}$, we first define the sample coverage in the total population as

$$ C = \sum_{i=1}^{A} p_i I\left(X_{i1} + X_{i2} + \ldots + X_{in} > 0\right), $$

which represents the total probabilities (or proportions) of the alleles observed in at least one of the samples. Using the concept of Turing [4], we can obtain the following estimator:

$$ \hat{C} = 1 - \frac{1}{n}\sum_{j=1}^{n} \frac{F_1(j)}{z_j}, $$

where $F_1(j)$ is the number of singletons that only appear in sample j but not in the other samples. Thus the probability $\bar{p}_{i+}$ can be estimated by $\tilde{\bar{p}}_{i+} = (\hat{C}/n)\sum_{j=1}^{n} X_{ij}/z_j$. Therefore, our proposed estimator of Shannon entropy in the total population is

$$ {}^1\hat{H}_T = -\sum_i \frac{\tilde{\bar{p}}_{i+} \log \tilde{\bar{p}}_{i+}}{1-\prod_{j=1}^{n}(1-\tilde{p}_{ij})^{z_j}} I(\sum_{j=1}^{n} X_{ij} > 0). $$

The empirical Shannon differentiation is calculated as $({}^1\hat{H}_T - {}^1\hat{H}_S)/\log n$.

Expected values under models

(1) *IAM expected in an isolated population*

From Eq. 3a of the main text, Shannon entropy and heterozygosity have a direct relationship under IAM:

$$ {}^1H = \psi(1/(1-{}^2H)) + 0.5772. $$

Substituting ${}^{2}H$ by its empirical estimate, then we can obtain IAM expected Shannon entropy.

(2) ***SMM expected in an isolated population***

Shannon entropy and heterozygosity have a direct relationship under SMM (Eq. 5b of the main text):

$$ {}^{1}H \approx \log\left(\frac{1+{}^{2}H-({}^{2}H)^{2}}{1-{}^{2}H}\right). $$

Substituting ${}^{2}H$ by its empirical estimate, then we can obtain IAM expected Shannon entropy.

(3) ***IAM-FIM expected values for the total population and subpopulation***

The IAM-FIM Shannon entropy of the total population can be directly computed from the total-population heterozygosity via a relationship under IAM (Eq. 3a of the main text):

$$ {}^{1}H_{T} = \psi(1/(1-{}^{2}H_{T})) + 0.5772. $$

Substituting ${}^{2}H_{T}$ by its empirical estimate, then we can obtain IAM-FIM expected Shannon entropy for the total population. For subpopulation Shannon entropy, we show that the subpopulation Shannon entropy can be written as a complicated function of total-population and subpopulation heterozygosities. Based on the formulas in Table 1 of the main text, we have

$$ \frac{1}{1-{}^{2}H_{T}} = \theta_{T} + 1 = 4Nn\mu + \frac{m^{*} + n\mu}{m^{*} + \mu}, $$

and

$$ {}^{2}H_{S} = 1 - \frac{4Nm^{*}/(\theta_{T}+1)+1}{4N(m^{*}+\mu)+1}. $$

These lead to

$$\frac{1-{}^2H_S}{1-{}^2H_T} = (\theta_T+1)(1-{}^2H_S) = \frac{4Nm^*+\theta_T+1}{4N(m^*+\mu)+1}$$

$$= \frac{1}{(m^*+\mu)}\frac{4Nm^*(m^*+\mu)+4Nn\mu(m^*+\mu)+(m^*+n\mu)}{4N(m^*+\mu)+1}$$

$$= \frac{1}{(m^*+\mu)}\frac{4N(m^*+\mu)(m^*+n\mu)+(m^*+n\mu)}{4N(m^*+\mu)+1} = \frac{m^*+n\mu}{m^*+\mu}.$$

Then we have

$$4N\mu = \frac{1}{n}\left(\frac{1}{1-{}^2H_T} - \frac{1-{}^2H_S}{1-{}^2H_T}\right) = \frac{1}{n}\frac{{}^2H_S}{(1-{}^2H_T)}. \tag{D5}$$

Further, from the approximation formula $[1+m^*/(n\mu)]^{-1} \approx \frac{({}^2H_T-{}^2H_S)}{1-{}^2H_S}\frac{n}{n-1}$ and the subpopulation heterozygosity (Table 1 of the main text) we obtain

$$\frac{1}{1-{}^2H_S} = \frac{4N(m^*+\mu)}{1+m^*/(n\mu)}+1 = 4N(m^*+\mu)\frac{({}^2H_T-{}^2H_S)}{1-{}^2H_S}\frac{n}{n-1}+1,$$

which leads to

$$4N(m^*+\mu) = \frac{n-1}{n}\frac{{}^2H_S}{({}^2H_T-{}^2H_S)}.$$

This formula and Eq. D5 then gives

$$4Nm^* = \frac{n-1}{n}\frac{{}^2H_S}{({}^2H_T-{}^2H_S)} - \frac{1}{n}\frac{{}^2H_S}{(1-{}^2H_T)}. \tag{D6}$$

Now both $4N(m^*+\mu)$ and $4Nm^*$ can be written as a function of total-population and subpopulation heterozygosities, the IAM-FIM expected subpopulation Shannon entropy (Eq. 7c in the main text) can then be expressed as

$$^1H_S = \psi[4N(m^*+\mu)+1] - \int_0^1 \psi(4Nm^*y+1)\theta_T(1-y)^{\theta_T-1}dy$$

$$= \psi\left(\frac{n-1}{n}\frac{{}^2H_S}{{}^2H_T-{}^2H_S}+1\right) - \int \psi\left\{\left[\frac{(n-1)}{n}\frac{{}^2H_S}{{}^2H_T-{}^2H_S} - \frac{{}^2H_S}{n(1-{}^2H_S)}\right]y+1\right\}\theta_T(1-y)^{\theta_T-1}dy. \tag{D7}$$

Therefore, given empirical estimates of the total-population and subpopulation

heterozygosities, numerically we can evaluate the IAM-FIM expected subpopulation Shannon entropy, and then the expected Shannon differentiation.

(4) ***SMM-FIM expected values for the total population and subpopulation***

Shannon entropy and heterozygosity for the total population under SMM-FIM follow the same relationship as that for an isolated population (Eq. 5b of the main text), i.e.,

$$ {}^1H_T \approx \log\left(\frac{1+{}^2H_T-({}^2H_T)^2}{1-{}^2H_T}\right) \; . $$

Substituting ${}^2H_T$ by its empirical estimate, then we can obtain SMM-FIM expected Shannon entropy for a subpopulation.

For subpopulation, first note the total- and subpopulation heterozygosities are functions of Nm^* and $N\mu$ (Eqs. 8a and 8b of the main text):

$$ {}^2H_T = 1-\frac{1}{\pi}\int_0^{\pi}\left(\frac{m^*/n\mu}{(1-\cos t)}+\frac{1}{n}\right)\left(4N(1-\cos t)\mu+\frac{m^*/n\mu}{(1-\cos t)}+1\right)^{-1} dt\,; \qquad \text{(D8)} $$

$$ {}^2H_S = 1-\frac{1}{\pi}\int_0^{\pi}\left(\frac{m^*/n\mu}{(1-\cos t)}+1\right)\left(4N(1-\cos t)\mu+\frac{m^*/n\mu}{(1-\cos t)}+1\right)^{-1} dt\,. \qquad \text{(D9)} $$

From the empirical heterozygosties, we can solve Nm^* and $N\mu$ from the above two equations. Then using the three relationships (see S3 Appendix): $\theta_T = [1/(1-{}^2H_T)^2-1]/2$, $\alpha_T = [1/(1-{}^2H_T)-1]/2$, and $\alpha_S = 4Nm^*\frac{{}^2H_T-{}^2H_S}{{}^2H_S}+4N\mu\frac{1-{}^2H_S}{{}^2H_S}-1$, to obtain approximate values of θ_T, α_T and α_S for given empirical estimates of total-population and subpopulation heterozygosities. Then we can obtain the SMM-FIM expected subpopulation Shannon entropy via the following formula (see Table 1 of the main text):

$$ {}^1H_S = \psi(4Nm^*+4N\mu+\alpha_S+1)-\int_0^1\frac{\psi(4Nm^*y+\alpha_S+1)}{B(\alpha_T+1,\theta_T)}y^{\alpha_T}(1-y)^{\theta_T-1}dy. \qquad \text{(D10)} $$

References

1. Chao A, Wang YT, Jost L. Entropy and the species accumulation curve: a novel entropy estimator via discovery rates of new species. Methods Ecol Evol. 2013; 4: 1091-1100.
2. Chao A, Shen T-J. Nonparametric estimation of Shannon's index of diversity when there are unseen species in sample. Environ Ecol Stat. 2003; 10: 429-443.
3. Horvitz DG, Thompson DJ. A generalization of sampling without replacement from a finite universe. J Am Stat Assoc. 1952; 47: 663-685.
4. Good IJ. The population frequencies of species and the estimation of population parameters. Biometrika. 1953; 40: 237-264.
