# Supplementary material for: Expected Shannon Entropy and Shannon Differentiation between Subpopulations for Neutral Genes under the Finite Island Model
Source: PLoS One. 2015 Jun 11;10(6):e0125471. doi: 10.1371/journal.pone.0125471 (PMC4465833; doi:10.1371/journal.pone.0125471)
Supplement: S5 Appendix — (PDF) [file pone.0125471.s005.pdf]

Supporting Information

Expected Shannon entropy and Shannon differentiation between subpopulations for neutral genes under the finite island model

Anne Chao, Lou Jost, T. C. Hsieh, K. H. Ma, William B. Sherwin, and Lee Ann Rollins

S5 Appendix. Dopamine receptor D4 (*DRD4*) and microsatellite data (3 loci) of four starling populations (Group 1-Group 4)

Table A. (*DRD4* data) Genotype frequency data for four groups of starling populations [1,2]

Genotype	Group 1	Group 2	Group 3	Group 4
1	8	9	36	12
2	54	3	70	14
3	2	0	89	13
4	1	0	3	0
5	23	6	11	2
6	22	11	15	7
7	10	2	2	2
8	15	1	14	8
9	3	0	31	13
10	1	9	79	48
11	1	0	0	0
12	2	0	0	0
13	1	8	10	5
14	1	1	4	3
15	0	1	0	0
16	1	1	23	8
17	0	0	7	2
18	1	0	22	8
19	0	0	16	4
20	0	0	1	0

21	0	0	1	0
22	0	0	18	7
23	0	0	17	3
24	0	0	2	0
25	0	0	2	4
26	0	0	2	0
27	0	0	2	1
28	0	0	1	1
29	0	0	1	0
30	0	0	1	0
31	0	0	1	0
32	0	0	1	0
33	0	0	1	0
34	0	0	3	7
35	0	0	0	1
36	0	0	0	1
37	0	0	0	1
38	0	0	0	1
Number of haplotypes	16	11	31	25
Sample size	146	52	486	176

Group 1: four localities {WMB, WJC, WMN, WCB} in Western Australia

Group 2: A single locality {WCO} in Condingup, Western Australia

Group 3: eight localities {SNU, SCR, SST, STB, SAS, SMA, SMC, SMN} in South Australia

Group 4: four localities {VIC, NSW, TAN, TAS} in Victoria, New South Wales and Tasmania

Table B. Microsatellite data for four groups of starling populations, numbered in order from west (Group 1) to east (Group 4). The localities included in each of the four groups are given in the footnotes of Table A [1,2]

(a) Allele frequency data in Locus Sta213 for four groups of starling populations

Allele	Group 1	Group 2	Group 3	Group 4
155	1	0	3	19
160	1	3	108	25
165	1	2	78	23
170	0	0	0	1
172	82	3	66	36
174	62	13	105	50
177	34	17	45	19
179	0	6	2	6
182	109	13	39	22
184	0	0	3	10
187	0	0	33	27
191	5	11	61	18
208	0	0	2	1
212	1	8	75	17
# of alleles	9	9	13	14
Sample size	296	76	620	274

(b) Allele frequency data in Locus Sta294 for four groups of starling populations

Allele	Group 1	Group 2	Group 3	Group 4
292	1	2	39	23
294	139	1	66	14
298	0	7	35	1
300	52	7	83	39
302	37	10	66	74
304	62	32	197	82
306	5	17	130	41
# of alleles	6	7	7	7
Sample size	296	76	616	274

(c) Allele frequency data in Locus Sta308 for four groups of starling populations

Allele	Group 1	Group 2	Group 3	Group 4
130	0	0	0	1
132	35	0	31	16
134	9	29	100	46
136	57	3	28	21
137	3	5	54	6
139	63	0	0	1
143	0	0	0	7
145	15	5	111	50
147	31	18	78	41
149	32	0	32	23
151	50	14	130	36
153	0	2	5	12
157	1	0	49	11
159	0	0	2	3
# of alleles	10	7	11	14
Sample size	296	76	620	274

Table C. Consistency of empirical data with SMM. Empirical and expected values for (a) single-population analysis under SMM, and for (b) total population, subpopulation and differentiation under SMM-FIM, based on the microsatellites for each subpopulation under SMM at locus Sta213. Data are shown in Table B. See Table 1 of the main text for the expected formulas and S4 Appendix for statistical methods to obtain empirical values. The proportional difference PD ≡ (expected value – estimated value)/expected value. All s.e. estimates were obtained by a bootstrap method based on 1000 resamples generated from the observed allele frequency distribution.

(a) Empirical and expected values by treating each of the four subpopulations as an isolated population following SMM for mutation

Method/Model	Measure	Subpopulation 1	Subpopulation 2	Subpopulation 3	Subpopulation 4
Empirical	Estimated Shannon $^1\hat{H}$	**1.4724**	**2.0617**	**2.2071**	**2.4301**
	(s.e.)	(0.0426)	(0.0695)	(0.0223)	(0.0339)
	Estimated heterozygosity $^2\hat{H}$	0.7327	0.8607	0.8788	0.9016
	(s.e.)	(0.0115)	(0.0151)	(0.0035)	(0.0055)
SMM expected#	Expected Shannon ^1H (Eq. 5b)	**1.4983**	**2.0844**	**2.2115**	**2.4036**
	(s.e.)	(0.0380)	(0.0906)	(0.0262)	(0.0524)
	Proportional difference (PD)	0.0173	0.0109	0.0020	-0.0111

#The expected parameters under SMM for the four subpopulations: $N\mu$ = (1.6249, 6.3170, 8.3838, 12.7796); see Eq. 4b. of the main text.

(b) Empirical and SMM-FIM expected values for total-population, subpopulation and differentiation measures at loci Sta213

Methods or assumptions	Measure	Total population	Subpopulation	Shannon Differentiation	Jost Differentiation	G_{ST}
Empirical	Estimated Shannon $^1\hat{H}$	**2.2655**	**2.0428**	**0.1606**		
	(s.e.)	(0.0222)	(0.0235)	(0.0159)		
	Estimated heterozygosity $^2\hat{H}$	0.8808	0.8435		0.3182	0.0443
	(s.e.)	(0.0038)	(0.0049)		(0.0314)	(0.0058)
SMM-FIM Expected#	Expected Shannon ^1H	**2.2246***	**2.0368§**	**0.1355**		
	(s.e.)	(0.0280)	(0.0277)	(0.0190)		
	Proportional difference	-0.0184	-0.0030	-0.1855		

# The expected parameters under SMM-FIM: $N\mu$ = 8.14, Nm = 9.68; see Eqs. D8 and D9 of S4 Appendix.

* Total population entropy value calculated from total population heterozygosity under SMM via Eq. 5b of the main text: $^1H_T \approx \log\{[1+{}^2H_T - ({}^2H_T)^2]/(1-{}^2H_T)\}$.

§ Subpopulation entropy is calculated from heterozygosity via a link described in S4 Appendix.

Table D. Consistency of empirical data with SMM. Empirical and expected values for (a) single-population analysis under SMM, and for (b) total population, subpopulation and differentiation under SMM-FIM, based on the microsatellites for each subpopulation under SMM at locus Sta294. Data are shown in Table B. See Table 1 of the main text for the expected formulas and S4 Appendix for statistical methods to obtain empirical values. The proportional difference PD ≡ (expected value – estimated value)/expected value. All s.e. estimates were obtained by a bootstrap method based on 1000 resamples generated from the observed allele frequency distribution.

(a) Empirical and expected values by treating each of the four subpopulations as an isolated population following SMM for mutation

Method/Model	Measure	Subpopu-lation 1	Subpopu-lation 2	Subpopu-lation 3	Subpopu-lation 4
Empirical	Estimated Shannon ${}^1\hat{H}$	**1.3460**	**1.6043**	**1.8420**	**1.6693**
	(s.e.)	(0.0393)	(0.0896)	(0.0226)	(0.0345)
	Estimated heterozygosity ${}^2\hat{H}$	0.6912	0.7474	0.8061	0.7881
	(s.e.)	(0.0177)	(0.0329)	(0.0077)	(0.0103)
SMM expected#	Expected Shannon ${}^1H$ (Eq. 5b)	**1.3684**	**1.5488**	**1.7858**	**1.7059**
	(s.e.)	(0.0530)	(0.1186)	(0.0367)	(0.0441)
	Proportional difference (PD)	0.0164	-0.0359	0.0009	0.0214

#The expected parameters under SMM for the four subpopulations: $N\mu$ = (1.1855, 1.8336, 3.2013, 2.6577); see Eq. 4b of the main text.

(b) Empirical and SMM-FIM expected values for total-population, subpopulation and differentiation measures at loci Sta294

Methods or assumptions	Measure	Total population	Subpopulation	Shannon Differentiation	Jost Differentiation	G_{ST}
Empirical	Estimated Shannon ${}^1\hat{H}$	**1.7677**	**1.6010**	**0.1203**		
	(s.e.)	(0.0217)	(0.0278)	(0.0142)		
	Estimated heterozygosity ${}^2\hat{H}$	0.8081	0.7582		0.2752	0.0658
	(s.e.)	(0.0068)	(0.0104)		(0.0285)	(0.0097)
SMM-FIM Expected#	Expected Shannon ${}^1H$	**1.7891***	**1.5886§**	**0.1446**		
	(s.e.)	(0.0308)	(0.0348)	(0.0161)		
	Proportional difference	0.0119	-0.0078	0.1679		

# The expected parameters under SMM-FIM: $N\mu$ = 3.05, Nm = 8.06; see Eqs. D8 and D9 of S4 Appendix.

* Total population entropy value calculated from total population heterozygosity under SMM via Eq. 5b of the main text: ${}^1H_T \approx \log\{[1+{}^2H_T-({}^2H_T)^2]/(1-{}^2H_T)\}$.

§ Subpopulation entropy is calculated from heterozygosity via a link described in S4 Appendix.

Table E. Consistency of empirical data with SMM. Empirical and expected values for (a) single-population analysis under SMM, and for (b) total population, subpopulation and differentiation under SMM-FIM, based on the microsatellites for each subpopulation under SMM at locus Sta308. Data are shown in Table B. See Table 1 of the main text for the expected formulas and S4 Appendix for statistical methods to obtain empirical values. The proportional difference PD ≡ (expected value – estimated value)/expected value. All s.e. estimates were obtained by a bootstrap method based on 1000 resamples generated from the observed allele frequency distribution.

(a) Empirical and expected values by treating each of the four subpopulations as an isolated population following SMM for mutation

Method/Model	Measure	Subpopulation 1	Subpopulation 2	Subpopulation 3	Subpopulation 4
Empirical	Estimated Shannon ${}^1\hat{H}$	**2.0161**	**1.6427**	**2.1120**	**2.2944**
	(s.e.)	(0.0348)	(0.0941)	(0.0243)	(0.0440)
	Estimated heterozygosity ${}^2\hat{H}$	0.8517	0.7635	0.8624	0.8809
	(s.e.)	(0.0067)	(0.0307)	(0.0047)	(0.0070)
SMM expected#	Expected Shannon ${}^1H$ (Eq. 5b)	**2.0276**	**1.6078**	**2.0957**	**2.2279**
	(s.e.)	(0.0397)	(0.1090)	(0.0310)	(0.0487)
	Proportional difference (PD)	0.0056	-0.0217	-0.0078	-0.0299

#The expected parameters under SMM for the four subpopulations: $N\mu$ = (5.5600, 2.1100, 6.4790, 8.6930); see Eq. 4b of the main text.

(b) Empirical and SMM-FIM expected values for total-population, subpopulation and differentiation measures at loci Sta308

Methods or assumptions	Measure	Total population	Subpopulation	Shannon Differentiation	Jost Differentiation	G_{ST}
Empirical	Estimated Shannon ${}^1\hat{H}$	**2.2512**	**2.0163**	**0.1695**		
	(s.e.)	(0.0213)	(0.0266)	(0.0138)		
	Estimated heterozygosity ${}^2\hat{H}$	0.8774	0.83965		0.3138	0.0449
	(s.e.)	(0.0037)	(0.0081)		(0.0347)	(0.0076)
SMM-FIM Expected#	Expected Shannon ${}^1H$	**2.1984***	**1.9948§**	**0.1469**		
	(s.e.)	(0.0266)	(0.0321)	(0.0176)		
	Proportional difference	-0.0240	-0.0108	-0.1535		

# The expected parameters under SMM-FIM: $N\mu$ = 7.74, Nm = 9.60; see Eqs. D8 and D9 of S4 Appendix.

* Total population entropy value calculated from total population heterozygosity under SMM via Eq. 5b of the main text: ${}^1H_T \approx \log\{[1+{}^2H_T-({}^2H_T)^2]/(1-{}^2H_T)\}$.

§ Subpopulation entropy is calculated from heterozygosity via a link described in S4 Appendix.

References

1. Rollins LA, Woolnough AP, Wilton AN, Sinclair R, Sherwin WB. Invasive species can't cover their tracks: using microsatellites to assist management of starling (Sturnus vulgaris) populations in Western Australia. Mol Ecol. 2009; 18: 1560-1573.
2. Rollins LA. A molecular investigation of dispersal, drift and selection to aid management of an invasion in progress. Thesis, The University of New South Wales. 2009.
