# Supplementary material for: Expected Shannon Entropy and Shannon Differentiation between Subpopulations for Neutral Genes under the Finite Island Model
Source: PLoS One. 2015 Jun 11;10(6):e0125471. doi: 10.1371/journal.pone.0125471 (PMC4465833; doi:10.1371/journal.pone.0125471)
Supplement: S6 Appendix — (PDF) [file pone.0125471.s006.pdf]

Supporting Information

Expected Shannon entropy and Shannon differentiation between subpopulations for neutral genes under the finite island model

Anne Chao, Lou Jost, T. C. Hsieh, K. H. Ma, William B. Sherwin, and Lee Ann Rollins

S6 Appendix. Two strong monotonicity properties for mutual information and Shannon differentiation measures

Assume there are n subpopulations and A alleles in the total population. Let x_{ik} be the allele frequency of the ith allele in the kth subpopulation, $i = 1, 2, \ldots, A$, $k = 1, 2, \ldots, n$. Some of the x_{ik} may be zero. In the main text, we assumed that all subpopulations are equally weighted, and we gave definitions for the mutual information and Shannon differentiation only for the equal-weight case; see Eq. 10 in the main text. Here we extend the definitions to the un-equal weight case. Suppose the weights for the subpopulations are $\{w_1, w_2, \ldots, w_n\}$; the weights can be equal weights, subpopulation sizes or any types of weights. The relative frequency of the ith allele in the kth subpopulation is $p_{ik} = x_{ik} / x_{+k}$, where $x_{+k} = \sum_{i=1}^{A} x_{ik}$ is the population size of the kth subpopulation.

Let $\bar{p}_{i+} = \sum_{k=1}^{n} w_k x_{ik} / x_{+k}$ be the relative frequency of allele i in the total population for the weights $\{w_1, w_2, \ldots, w_n\}$. Then the Shannon entropy for the total population is defined as

$$ {}^1H_T = -\sum_{i=1}^{A} \bar{p}_{i+} \log \bar{p}_{i+} . $$

The Shannon entropy for a subpopulation under the same weights is

$$ {}^1H_S = -\sum_{k=1}^{n} w_k \sum_{i=1}^{A} p_{ik} \log p_{ik} . $$

The mutual information for the weights $\{w_1, w_2, \ldots, w_n\}$ can be expressed as

$$ {}^1H_T - {}^1H_S = -\sum_{i=1}^{A} \bar{p}_{i+} \log \bar{p}_{i+} - \sum_{k=1}^{n} w_k \sum_{i=1}^{A} p_{ik} \log p_{ik} \ . $$

This mutual information ranges from 0 to the Shannon entropy of the weights. The Shannon differentiation for the weights $\{w_1, w_2, ..., w_n\}$ is the standardized mutual information:

$$ \text{Shannon differentiation} = 1 - C_{1n} = \frac{{}^1H_T - {}^1H_S}{-\sum_{i=1}^{n} w_i \log w_i} . \quad \text{(F1)} $$

This measure quantifies the differentiation among the n sets of allele relative frequencies $\{(p_{1k}, p_{2k}, ..., p_{Ak}); k = 1, 2, ..., n\}$ with weight w_k for the kth subpopulation set. When all weights are equal ($w_1 = w_2 = ... = w_n = 1/n$), the above Shannon differentiation reduces to Eq. 10 of the main text.

(A) Proof of a strong monotonicity property for mutual information and Shannon differentiation: the two measures always increase when some copies of an allele that is shared between two or more subpopulations are replaced by copies of an unshared allele.

Assume that the last allele (allele A) is a shared allele and its frequency is $x_{A1} = x > 0$. Let a ($0 \le a \le x$) out of the x copies of this shared allele in subpopulation 1 be replaced by copies of a new unshared allele (allele A+1). Then the allele frequency set in the new subpopulation 1 (with A+1 alleles) becomes

$$ \{x_{11}, x_{21}, ..., x - a, a\} . $$

Let $R = \sum_{k=2}^{n} w_k \frac{x_{Ak}}{x_{+k}}$ and define $\log 0 \equiv 0$. Here $R > 0$ because allele A is a shared allele.

Then for the new subpopulation 1 and the subpopulations 2, 3, …, n, the mutual information becomes

$$ {}^1H_{T,new} - {}^1H_{S,new} = -\sum_{i=1}^{A-1} \bar{p}_{i+} \log \bar{p}_{i+} - (w_1 \frac{x-a}{x_{+1}} + R) \log(w_1 \frac{x-a}{x_{+1}} + R) - w_1 \frac{a}{x_{+1}} \log(w_1 \frac{a}{x_{+1}}) $$

$$ + w_1 \sum_{i=1}^{A-1} \frac{x_{i1}}{x_{+1}} \log \frac{x_{i1}}{x_{+1}} + w_1 \frac{x-a}{x_{+1}} \log \frac{x-a}{x_{+1}} + w_1 \frac{a}{x_{+1}} \log \frac{a}{x_{+1}} + \sum_{k=2}^{n} w_k \sum_{i=1}^{A} \frac{x_{ik}}{x_{+k}} \log \frac{x_{ik}}{x_{+k}} . $$

Regard this as a function of a and define

$$g(a) = {}^1H_{T,new} - {}^1H_{S,new}, \quad 0 \le a \le x.$$

Note that for $a = 0$, we have $g(0) = {}^1H_T - {}^1H_S$, the mutual information for the original subpopulations 1, 2, …, n. We next show for any a between 0 and x, the following derivative is always positive.

$$g'(a) = \frac{w_1}{x_{+1}} \log(w_1 \frac{x-a}{x_{+1}} + R) + \frac{w_1}{x_{+1}} - \frac{w_1}{x_{+1}} \log(w_1 \frac{a}{x_{+1}}) - \frac{w_1}{x_{+1}}$$

$$- \frac{w_1}{x_{+1}} \log \frac{x-a}{x_{+1}} - \frac{w_1}{x_{+1}} + \frac{w_1}{x_{+1}} \log \frac{a}{x_{+1}} + \frac{w_1}{x_{+1}}$$

$$= \frac{w_1}{x_{+1}} \log(w_1 \frac{x-a}{x_{+1}} + R) - \frac{w_1}{x_{+1}} \log w_1 - \frac{w_1}{x_{+1}} \log \frac{x-a}{x_{+1}}$$

$$= \frac{w_1}{x_{+1}} \log\left[\frac{(x-a) + (R/w_1)x_{+1}}{x-a}\right] > 0.$$

This implies that both the mutual information and Shannon differentiation measure are increasing with the added abundance of the new allele. Moreover, we have proved our monotonicity property for mutual information, i.e.,

$$g(a) = {}^1H_{T,new} - {}^1H_{S,new} > g(0) = {}^1H_T - {}^1H_S.$$

We provide an example to show that G_{ST} and Jost's D do not satisfy the above monotonicity property. Assume the allele frequency distributions for subpopulations 1 and 2 are given in the following table:

Allele	Subpo-pulation 1	Subpo-pulation 2	New sub-population 1	Subpo Pulation 2
1	7	1	7	1
2	4	4	4	4
3	0	2	0	2
4	0	13	0	13
5	10	1	9	1
6			1	0
Column sum	21	21	21	21

Suppose one of 10 copies of allele 5 becomes an unshared allele (allele 6). Then we have the following table for the case where the subpopulations have equal weights:

Subpopulations	G_{ST}	Jost's D	Shannon differentiation $(1-C_{1n})$
Subpopulation 1 vs. subpopulation 2	0.216	0.815	0.591
New subpopulation 1 vs. subpopulation 2	0.201	0.811	0.594

Thus, G_{ST} and Jost's D fail to satisfy the above monotonicity property. In this example, only Shannon differentiation measure satisfies the property. Both G_{ST} and Jost's D weight alleles according to the square of their frequencies, so that the most abundant alleles dominate these measures. In this example, the most abundant allele in each subpopulation is almost absent in the other subpopulation, resulting in high differentiation even though other less-abundant alleles are shared between both subpopulations. When the frequency of the most abundant allele in subpopulation 1 is reduced, the differentiation measure gives it much less weight than before, and gives more weight to the shared alleles. In applications where the dynamics of some processes depend on the *squares* of the allele frequencies, this failure makes sense, but in other applications, it is better to have a measure that does not violate this very natural monotonicity condition.

(B) The monotonicity property proposed by Jost et al. [1] when the weights are predetermined and fixed: mutual information and Shannon differentiation are always non-decreasing any time a new allele is added to any single subpopulation, with any abundance.

Suppose we add a new allele (allele A+1) with abundance x into subpopulation 1. Then the frequency set in the new subpopulation 1 is

$$\{x_{11}, x_{21}, ..., x_{A1}, x\}.$$

Define $\lambda = x/(x_{+1} + x)$, which is the relative frequency of allele A+1 in the new subpopulation 1. The allele relative frequencies in the new subpopulation 1 are changed to

$$\{(1-\lambda)p_{11}, (1-\lambda)p_{21}, ..., (1-\lambda)p_{A1}, \lambda\}.$$

Let $\overline{p}_{i+}^{*} = \sum_{k=2}^{n} w_k p_{ik}$. Based on any pre-determined weights $\{w_1, w_2, ..., w_n\}$ which are fixed in advance and unchanged by the addition of the new allele, the mutual information for the new subpopulation 1 and subpopulations 2, 3, …, n becomes

$$
\begin{aligned}
{}^1H_{T,new} - {}^1H_{S,new} &= -\sum_{i=1}^{A}[(1-\lambda)w_1 p_{i1} + \overline{p}_{i+}^{*}]\log[(1-\lambda)w_1 p_{i1} + \overline{p}_{i+}^{*}] - w_1\lambda\log(w_1\lambda) \\
&\quad + w_1\sum_{i=1}^{A}[(1-\lambda)p_{i1}\log[(1-\lambda)p_{i1}] + w_1\lambda\log\lambda + \sum_{k=2}^{n} w_k \sum_{i=1}^{A} p_{ik}\log p_{ik}.
\end{aligned}
$$

Regard this as a function of λ and define

$$f(\lambda) = {}^1H_{T,new} - {}^1H_{S,new}, \quad \lambda \geq 0.$$

Note that for $\lambda = 0$, we have $f(0) = {}^1H_T - {}^1H_S$, the mutual information for the original subpopulations 1, 2, …, n. We show for any $\lambda \geq 0$, the following derivative is always non-negative.

$$f'(\lambda)=\sum_{i=1}^{A}w_1p_{i1}\log[(1-\lambda)w_1p_{i1}+\overline{p}_{i+}^{*}]+\sum_{i=1}^{A}w_1p_{i1}-w_1\log(w_1\lambda)-w_1$$

$$-w_1\sum_{i=1}^{A}p_{i1}\log[(1-\lambda)p_{i1}]-w_1\sum_{i=1}^{A}p_{i1}+w_1\log\lambda+w_1$$

$$=\sum_{i=1}^{A}w_1p_{i1}\log[(1-\lambda)w_1p_{i1}+\overline{p}_{i+}^{*}]-w_1\log w_1-w_1\sum_{i=1}^{A}p_{i1}\log[(1-\lambda)p_{i1}]$$

$$=\sum_{i=1}^{A}w_1p_{i1}\left(\log\left[\frac{(1-\lambda)w_1p_{i1}+\overline{p}_{i+}^{*}}{w_1}\right]+\log w_1\right)-w_1\log w_1-w_1\sum_{i=1}^{A}p_{i1}\log[(1-\lambda)p_{i1}]$$

$$=\sum_{i=1}^{A}w_1p_{i1}\log\left[\frac{(1-\lambda)w_1p_{i1}+\overline{p}_{i+}^{*}}{w_1}\right]-w_1\sum_{i=1}^{A}p_{i1}\log[(1-\lambda)p_{i1}]$$

$$=\sum_{i=1}^{A}w_1p_{i1}\log\left[\frac{(1-\lambda)p_{i1}+(\overline{p}_{i+}^{*}/w_1)}{(1-\lambda)p_{i1}}\right]\geq 0.$$

The above derivative is zero (i.e., mutual information remains the same) if and only if all alleles in subpopulation 1 (the subpopulation to which the new allele is added) are unshared. The above derivation reveals that the mutual information and Shannon differentiation both are non-decreasing with the added relative abundance λ (and thus with the raw abundance x) of the new allele. We have also proved this monotonicity property for the mutual information:

$$f(\lambda)={}^{1}H_{T,new}-{}^{1}H_{S,new}\geq f(0)={}^{1}H_{T}-{}^{1}H_{S}.$$

We give a simple example to show that G_{ST} and Jost's D do not satisfy the above monotonicity property. Assume the allele frequency distributions for subpopulations 1 and 2 are given in the following table:

Allele	Subpopulation 1	Subpopulation 2	New subpopulation 1	Sub-population 2
1	5	5	5	5
2	1	5	1	5
3	1	5	1	5
4	1	5	1	5
5			1	0

Suppose a new allele (allele 5) is added into subpopulation 1. Then we have the following table for the case where the subpopulations have equal weights:

Subpopulations	G_{ST}	Jost's D	Shannon differentiation ($1-C_{1n}$)
Subpopulation 1 vs. subpopulation 2	0.067	0.273	0.106
New subpopulation 1 vs. subpopulation 2	0.055	0.269	0.158

This table shows that only Shannon differentiation measure satisfies the property of monotonicity.

In the above proof, we assume that the weights are fixed in advance and are not affected by the addition of the new allele. Therefore, the weights are not functions of λ (and not function of the raw abundance x). However, if the weights are proportional to subpopulation sizes, then the weights after the addition of a new allele become a function of λ and x. In this case, the strong monotonicity property (B) is modified to the following.

(B) When the weights are proportional to subpopulation sizes, Shannon differentiation is always non-decreasing any time a new allele is added to a single subpopulation, with any abundance. However, the mutual information fails to satisfy this monotonicity property.*

As with the proof in (B), suppose we add a new allele (allele A+1) with abundance x into subpopulation 1. The frequency set in the new subpopulation 1 becomes $\{x_{11}, x_{21}, ..., x_{A1}, x\}$. In this case, it is more transparent to express the mutual information and Shannon differentiation as functions of x directly and take a derivative with respect to x. Notice that the mutual information for the new subpopulation 1 and subpopulations 2, 3, …, n can be expressed as

$$ {}^{1}H_{T,new} - {}^{1}H_{S,new} = -\sum_{i=1}^{A} \frac{x_{i+}}{x_{++}+x} \log\left(\frac{x_{i+}}{x_{++}+x} \right) - \frac{x}{x_{++}+x} \log\left(\frac{x}{x_{++}+x} \right) $$

$$ + \sum_{i=1}^{A} \frac{x_{i1}}{x_{++}+x} \log\left(\frac{x_{i1}}{x_{+1}+x} \right) + \frac{x}{x_{++}+x} \log\left(\frac{x}{x_{+1}+x} \right) $$

$$ + \sum_{k=2}^{n} \sum_{i=1}^{A} \frac{x_{ik}}{x_{++}+x} \log\left(\frac{x_{ik}}{x_{+k}} \right). $$

When the weights are proportional to subpopulation sizes, the weights for the new subpopulation 1 and subpopulations 2, 3, …, n is

$$ H_{w,new} = -\sum_{k=1}^{n} w_k \log w_k = -\frac{x_{+1}+x}{x_{++}+x} \log\left(\frac{x_{+1}+x}{x_{++}+x} \right) - \sum_{k=2}^{n} \frac{x_{+k}}{x_{++}+x} \log\left(\frac{x_{+k}}{x_{++}+x} \right). $$

Shannon differentiation is defined as

$$ h(x) = \frac{{}^{1}H_{T,new} - {}^{1}H_{S,new}}{H_{w,new}} = \frac{h_1(x)}{h_2(x)}, \quad x \geq 0, $$

where

$$ h_1(x) = -\sum_{i=1}^{A} x_{i+} \log\left(\frac{x_{i+}}{x_{++}+x} \right) - x \log\left(\frac{x}{x_{++}+x} \right) $$

$$ + \sum_{i=1}^{A} x_{i1} \log\left(\frac{x_{i1}}{x_{+1}+x} \right) + x \log\left(\frac{x}{x_{+1}+x} \right) + \sum_{k=2}^{n} \sum_{i=1}^{A} x_{ik} \log\left(\frac{x_{ik}}{x_{+k}} \right), $$

and

$$ h_2(x) = -(x_{+1}+x) \log\left(\frac{x_{+1}+x}{x_{++}+x} \right) - \sum_{k=2}^{n} x_{+k} \log\left(\frac{x_{+k}}{x_{++}+x} \right). $$

Then the derivative of Shannon differentiation is

$$ h'(x) = \frac{h_1'(x) h_2(x) - h_1(x) h_2'(x)}{[h_2(x)]^2}. $$

The derivatives of $h_1(x)$ and $h_2(x)$ turns out to be identical, i.e.,

$$ h_1'(x) = h_2'(x) = \log\left(\frac{x_{++}+x}{x_{+1}+x} \right). $$

This leads to

$$h'(x) = \frac{[H_w - ({}^1H_{T,new} - {}^1H_{S,new})]}{[h_2(x)]^2}(x_{++} + x)\log\left(\frac{x_{++} + x}{x_{+1} + x}\right) \geq 0 .$$

The last inequality follows from the fact that the maximum value of the mutual information is equal to the Shannon entropy of the weights, i.e., $H_w - ({}^1H_{T,new} - {}^1H_{S,new}) \geq 0$. It becomes an equality if all alleles are unshared. Thus, Shannon differentiation is non-decreasing with the added abundance x. Also, note that for $x = 0$, we have $h(0) = ({}^1H_T - {}^1H_S)/H_w$, the Shannon differentiation for the original subpopulations 1, 2, …, n. We thus complete the proof of this monotonicity property. However, the mutual information does not satisfy this strong monotonicity property. This can be readily seen by considering the special case that all alleles are unshared because in this special case the mutual information reduces to Shannon entropy of the weights. It is well known that Shannon entropy is reduced if the added abundance of a new allele is sufficiently large.

Reference

1. Jost L, DeVries P, Walla T, Greeney H, Chao A, Ricotta C. Partitioning diversity for conservation analyses. Divers Distrib. 2010; 16: 65-76.
